# Is Generalization General? Differences Between Predictive and Category Learning Reflect Knowledge of “Cognitive Kinds”

**DOI:** 10.1162/OPMI.a.368

**Published:** 2026-07-17

**Authors:** Jessica C. Lee, Andy J. Wills, René Schlegelmilch

**Affiliations:** University of Sydney, Sydney, Australia; University of New South Wales, Sydney, Australia; University of Plymouth, Plymouth, UK; University of Bremen, Bremen, Germany

**Keywords:** category learning, associative learning, generalization, predictive learning

## Abstract

Shepard (1987) observed that generalization gradients were invariant between species and stimulus dimensions, and proposed that these universal properties reflect knowledge of “natural kinds” that exist in the world. Here, we test a natural extension of Shepard’s ideas, by examining whether generalization differs between different types of learned associations. To do so, we leverage generalization as a tool to diagnose the underlying content of learning in two domains (predictive learning and category learning), while equating stimuli and procedural details between tasks. Experiment 1 showed that empirical differences in generalization gradients between domains can be largely explained by differences in the testing procedures. When these were equated, the underlying generalization function was largely similar between predictive and category learning. The results of Experiment 2 were more complex, revealing some differences between domains which may be explained by differences in the representation of outcomes and categories. We conclude that the similarities between learning domains (underlying gradient shape and response to experimental manipulations) provide support for Shepard’s (1987) proposal that generalization exhibits universal properties, and propose that the differences between learning domains reflect knowledge about “cognitive kinds”; categories are created to be mutually exclusive while outcomes are not.

## INTRODUCTION

Generalization is an inductive problem where a learner is tasked with inferring whether a stimulus leads to some consequential outcome, using their knowledge of other stimuli that are known to lead to that same outcome. Typically, learned responses to novel stimuli decrease according to the degree of dissimilarity to known stimuli, forming a *generalization gradient*. A seminal paper by Shepard (1987) proposed that generalization should be Psychology’s first law since re-scaling gradients of generalization according to psychological (as opposed to physical) distance between stimuli resulted in an exponential function that was invariant across stimulus dimensions and species. Shepard (1987) suggested that “these regularities reflect universal principles of natural kinds” (p. 1323), claiming that the observed invariances occur because consequential stimuli (stimuli that lead to important outcomes, e.g., food rewards, illness) have particular properties in the world which have been internalized through the course of evolution. Shepard’s work was groundbreaking because he demonstrated that behavior was lawful, and also proposed a theory which explained why this should be the case (Navarro, 2021). In this study, we test a natural extension of Shepard’s ideas. Specifically, we ask whether generalization is itself general, by testing whether generalization in the original predictive learning domain, differs from that of another domain involving learned associations; category learning (see also Gluck & Bower, 1988).

Prediction and categorization are both cases where humans learn how stimuli predict consequential outcomes and make judgments about novel stimuli. Predictive learning involves learning of predictive associations between stimuli and outcomes (e.g., Alloy & Abramson, 1979; Dickinson et al., 1984), while category learning involves learning of categorical associations between stimuli and category labels (e.g., Ashby & Maddox, 2005). In both domains, the content of learning is assessed by measuring judgments of novel stimuli of varying similarity to the learned stimuli in a generalization test, often without corrective feedback (Lee et al., 2022). The shape of the resulting generalization gradient is often used to infer what participants have learned. Although there are obvious differences in the semantic properties of a categorical and predictive association (e.g., an assumed temporal relationship between stimuli and outcomes in predictive learning), the tasks are similar in that they both involve learning of associations between stimuli and representations of events^1^. Given these similarities, generalization of predictive learning and category learning may have similar functional properties.

Comparison between these domains however, is not straightforward. Despite both predictive and category learning having origins in animal classical conditioning (Dickinson et al., 1984; Gluck & Bower, 1988; Mackintosh, 1995; Urcuioli et al., 2014), research in these domains has mostly proceeded separately, making a direct comparison with existing literature difficult. There are some phenomena that have been investigated in both domains such as acquired equivalence/distinctiveness (Honey & Hall, 1989), and peak shift (Hanson, 1959; Purtle, 1973), but these phenomena are often assumed to be functionally equivalent between domains without any formal testing. The shapes of resulting gradients from these domains also seem to differ in important ways. Anecdotally, the literature seems to indicate that gradients from differential predictive learning (where one stimulus leads to one outcome and another stimulus leads to the absence of that outcome) tend to be more Gaussian-like (e.g., Lee et al., 2018), while gradients from simple category learning (one stimulus per category) tend to be more sigmoidal (e.g., Lee & Livesey, 2018; Livesey & McLaren, 2009). At face value, this suggests that there may be a difference in mechanisms between learning domains, perhaps with more rule- or relation-based generalization in category learning compared to predictive learning. However, in order to make this assessment, it is necessary to control for procedural features that typically differ between tasks.

This point is illustrated elegantly in a study conducted by Hendrickson et al. (2019), who showed how seemingly minor task differences can have dramatic consequences on generalization behavior and subsequent theoretical conclusions. Hendrickson et al. (2019) compared generalization in groups of participants learning about stimuli (exemplars) from one (category A) or two categories (category A vs. B). They were interested in testing how the sample size of the category affected generalization, and therefore provided either 4 or 12 exemplars from the target category (A). Intriguingly, they found that increasing sample size decreased generalization (i.e., tightened the generalization gradient of target category A) when participants learned about one category, but increased generalization (i.e., broadened the generalization gradient of target category A) when participants learned about two categories.

**Figure 1.**
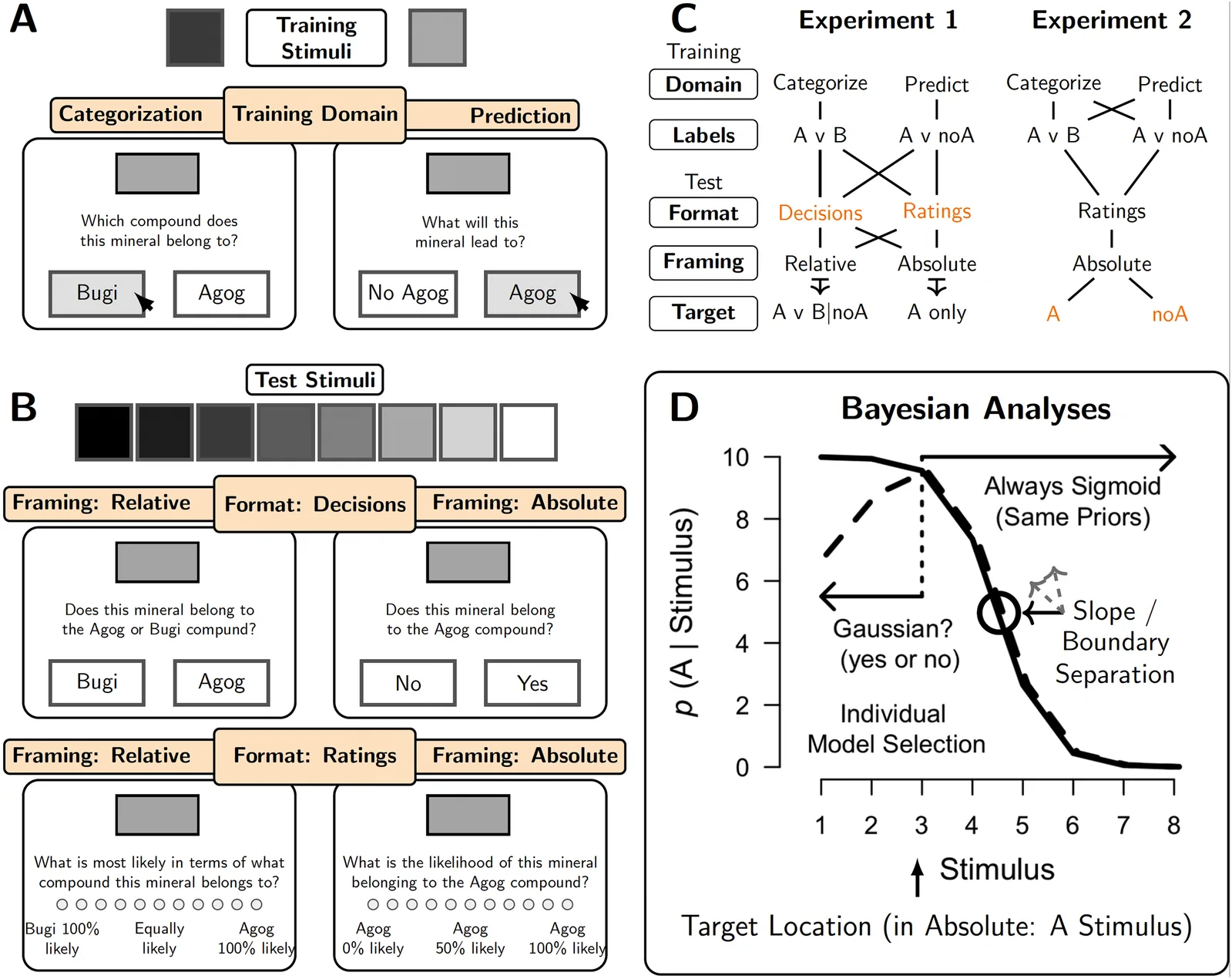
(A) Depiction of a training trial for each learning Domain (categorization vs. prediction). Note that the outcomes (A/B or A/noA) were fixed for each domain in Experiment 1 but manipulated between domains in Experiment 2. (B) Depiction of a generalization test trial for each Format (decisions vs. ratings) and Framing (relative vs. absolute) condition. Note that Format and Framing were manipulated in Experiment 1 but only absolute ratings were used in Experiment 2. (C) List of factors manipulated in each experiment. Factors highlighted in orange reflect within-participant conditions (with order counterbalanced), between-participant manipulations are in black text. (D). Schematic of Bayesian analysis method and key parameters. Slope: indexing response determinism (gradient steepness) between the training stimuli, *p*(Gaussian): indexing classification of gradients as Gaussian (decreasing beyond the training stimulus [position 3]) or sigmoid (increasing beyond the training stimulus), and midpoint: (location where the gradient reaches 50% on the response scale).

Further, these opposing results were each consistent with well-established theories of category learning employing very different mechanisms. Nosofsky’s Generalized Context Model (Nosofsky, 1986) assumes that subjects store exemplars from each category, and compute the similarity of new exemplars to these stored exemplars. The model predicts broadening of generalization since the computed similarity to stored exemplars tends to increase as more exemplars from that category are observed. In contrast, Tenenbaum and Griffith’s Bayesian theory (Tenenbaum & Griffiths, 2001) predicts tightening of generalization because additional exemplars increase confidence in the smallest category boundary that covers the observed exemplars (i.e., the size principle). The intuition behind this prediction is that if the category were broader, then exemplars outside of the category boundary should have been observed. Since Hendrickson et al. (2019) equated the superficial aspects of the tasks (e.g., stimuli, response measure), they claimed that the observed differences must represent systematic differences between the psychological processes engaged, and that generalizing from one category is a fundamentally different inductive problem than generalizing from two categories. In our study, we take a similar methodological approach to control for procedural differences (e.g., stimuli, testing format, see Figure 1A–C) in order to compare generalization between predictive and category learning domains.

### The Current Study

The aim of this study was to test whether generalization exhibits universal characteristics as proposed by Shepard (1987). This aim was assessed in two ways, by testing (1) whether the underlying generalization gradient differs between predictive and category learning domains (similar to Shepard, 1987), and (2) whether generalization between learning domains is affected in the same way by experimental manipulations (similar to Hendrickson et al., 2019). To the best of our knowledge, a direct comparison has not been made between predictive and category learning domains. This work follows other meta-theoretic attempts to bridge theoretical and methodological traditions between research domains (Hendrickson et al., 2019; Lee et al., 2025; Leising et al., 2025; Schlegelmilch, 2025) and heeds the call from researchers highlighting the importance of uncovering “new general principles that apply across fields and domains” for scientific progress (Shiffrin et al., 2026). We expand on each of our sub-aims below.

#### Underlying Gradient Shape.

Similar to Shepard (1987), we want to know whether there are similarities in the underlying generalization functions across domains. Unlike Shepard’s (1987) theory which was specifically formulated to describe generalization from a single stimulus leading to a consequential outcome (S+), we are interested in the case where participants learn about two stimuli with different consequences (e.g., S+ [leads to the outcome] and S− [leads to no outcome]). This discrimination learning scenario introduces complexity because as Shepard (1987) himself noted, “differential reinforcement could shape the generalization function…into a wide variety of forms.” (p. 1322).

Fortunately, the literature suggests that there are only two primary forms of generalization with simple discrimination learning. Generalization gradients that decrease as the test stimuli become more and more dissimilar from the training (i.e., familiar) stimulus have usually been taken to indicate similarity- or feature-based generalization. A Gaussian-shaped gradient is predicted when generalization is based on feature overlap (i.e., stimulus similarity) between stimuli (a Gaussian-like gradient is also predicted when noise is added to an exponential, Shepard, 1987). In contrast, gradients that are sigmoidal in shape or increase monotonically beyond the training stimuli, have usually been interpreted as evidence of rule-based or relational generalization (Lee et al., 2018; Lee & Livesey, 2018; Lee & Schlegelmilch, 2025; Livesey & McLaren, 2009; McLaren et al., 2014; Wills & Mackintosh, 1998). This sigmoidal gradient shape occurs when participants map a relational feature of the stimuli (e.g., S+ is slightly darker than S−) to the likelihood of the outcome, which should produce maximal predictions of the outcome for stimuli at the extreme end of the test dimension (e.g., the darkest stimulus), rather than the stimulus they have been trained with (S+).

Both of these generalization gradients have been reported in predictive and category learning (Lee et al., 2018; Lee & Livesey, 2018; Livesey & McLaren, 2009; Pothos & Wills, 2011; Rouder & Ratcliff, 2006; Schlegelmilch et al., 2022; Wills et al., 2000), and mixtures of these gradients can even be found within a single experimental condition (Lee et al., 2018; Lee, Lovibond, et al., 2021; Lovibond et al., 2020). Notably, gradient shape seems to show good correspondence to verbal reports about the content of learning (abstract rules vs. features/similarity, Lee et al., 2024). For our purposes, these individual differences demand a novel measurement approach capable of accounting for mixtures of sigmoidal and Gaussian-like gradients. This mixture also means that our research question involves a comparison of quantitative features (e.g., steepness) as well as qualitative features (e.g, shape) between predictive and categorical gradients.

#### Procedural Manipulations.

Our second approach to our research question is to test whether categorical and predictive generalization are affected in the same way by experimental manipulations. Following the logic of Hendrickson et al. (2019), if generalization in predictive and category learning domains were differentially affected by our manipulations, then this would suggest that there is a fundamental difference in the psychological process being studied in each task. Experiment 1 manipulated features of the testing procedure that typically differ between predictive and category learning, while Experiment 2 manipulated features of the training procedure (see Figure 1C).

In Experiment 1, we manipulated two features of the *testing* procedure that typically differ between learning domains. In category learning, researchers typically assess generalization over repeated binary decisions to calculate response probabilities (p(category A or B); e.g., Schlegelmilch et al., 2022). This judgment requires the integration of the relative likelihood of both categories in order to produce a binary decision. In predictive learning, however, researchers typically measure continuous probability ratings (i.e., outcome expectancy) and present each stimulus once to minimize potential extinction over repeated assessments (e.g., Lee et al., 2018, 2022) and/or unsupervised learning during test (see Livesey & McLaren, 2009). These testing procedures can be decomposed into two factors, which we term: *Format* (whether participants make continuous *Ratings* or binary *Decisions* about the predicted outcome), and *Framing* (whether the judgments ask about a single outcome [*Absolute*] or require integration of both outcomes [*Relative*]) (see Figure 1B).

We predicted that the apparent differences in gradient shape between domains might be explained by differences in testing procedure. Specifically, we hypothesized that the features associated with category learning (binary decisions and relative judgments) would produce more deterministic and rule-like gradients. This would be seen in steeper slopes between the training stimuli (see Figure 1D), as well as more sigmoidal-shaped gradients (see Gaussian vs. Sigmoid in Figure 1D; e.g., Hendrickson et al., 2019; Lee et al., 2018; Rouder & Ratcliff, 2006; Schlegelmilch et al., 2022). This is because decisions are often assumed to induce more response determinism than ratings, typically captured by modeling parameters of response scaling (see Krefeld-Schwalb et al., 2022; Pothos & Wills, 2011). Our hypothesis regarding Framing effects was based on work by Wills et al. (2000), who found that removing one outcome from the response options during test induced a qualitative change in participants’ response rules for the remaining outcome(s). In line with their arguments, we assume that Absolute judgments use information about a single outcome only, while Relative judgments involve integration from both categories in a ratio rule (Luce, 1959). In summary, we predicted steeper, more sigmoidal gradients for Relative judgments (compared to Absolute), as well as for binary Decisions (compared to Ratings).

In Experiment 2, we manipulated features of the *training* procedure, which also commonly differs between predictive and category learning. Category learning often involves sorting stimuli into one of two categories (category A or category B), while predictive learning involves learning about a single outcome that is either present or absent (outcome or no outcome). Therefore in Experiment 2, participants either learned about one outcome that was present or absent (AvnoA), or two outcomes (AvB). During test, we asked participants to judge the probability of A and noA outcomes in separate test phases.

In both experiments, the key manipulation was learning Domain: whether participants learned predictive associations (Prediction) of stimuli ‘leading to’ outcomes or categorical associations (Categorization) of stimuli ‘belonging to’ categories (see Figure 1A). For both experiments we hypothesized Null results for the Domain factor after controlling for the above-described manipulated task features. We also hypothesized that Domain would not interact with our experimental manipulations. These null results would support the idea that generalization exhibits universal characteristics as proposed by Shepard (1987). To foreshadow the results, we did obtain some effects of Domain, which led us to reflect on whether generalization about categories should in fact be similar to that of natural kinds (i.e., consequential outcomes).

## METHODS

### Participants

We recruited *N* = 309 English-speaking participants on Prolific Academic (age *M* = 39.77, *SD* = 12.99, 179 female, 119 male, 9 non-binary, 1 undisclosed) for Experiment 1, and *N* = 348 for Experiment 2 (age *M* = 39.07, *SD* = 12.91, 170 female, 170 male, 3 non-binary, 5 undisclosed). Participants were randomly allocated into conditions and we continued testing until we reached the preregistered minimum of 30 per between-subject design cell (Exp. 1. Domain: C = categorization vs. P = prediction, Framing: A = Absolute vs. R = Relative, Test Order: D = decisions first vs. R = ratings first: *N*_*CAD*_ = 33, *N*_*CAR*_ = 41, *N*_*PAR*_ = 38, *N*_*CRD*_ = 47, *N*_*CRR*_ = 35, *N*_*PRD*_ = 31, *N*_*PRR*_ = 46; Exp. 2. Domain: C = categorization vs. P = prediction, Training Labels: T = Two labels [A/B], O = One label [A/noA], Test Order: A = A ratings first vs. N = No A ratings first: *N*_*CTA*_ = 48, *N*_*CTN*_ = 46, *N*_*PTA*_ = 52, *N*_*PTN*_ = 34, *N*_*COA*_ = 44, *N*_*CON*_ = 40, *N*_*POA*_ = 46, *N*_*PON*_ = 38).

### Exclusion Criteria

We excluded participants who failed the instruction check more than twice (Exp. 1: *N* = 5; Exp. 2: *N* = 45), and who made errors in the last six training trials (Exp. 1: *N* = 12; Exp. 2: *N* = 15). We also allowed participants to opt out from the data analysis (e.g., due to distractions) at the end of the experiment (Exp. 1: *N* = 16; Exp. 2: *N* = 63). After these criteria were applied, 277 participants remained in Experiment 1 (over design cells: *N*_min_ = 32, *N*_max_ = 40), and 245 in Experiment 2 (30 participants in each design cell).

### Materials and Stimuli

The study was programmed with jsPsych (de Leeuw, 2015), and run online using the Prolific recruitment platform. The stimuli were greyscale rectangles presented on screen (200 px × 200 px), and the outcome labels consisted of either text labels “Agog” and “No Agog” (or “Not Agog” for Category Learning) or “Bugi”, depending on whether participants were allocated to AvnoA or AvB outcomes during training (see Figure 1).

### Procedure

The studies were approved by the University of New South Wales Human Research Ethics Advisory Panel and pre-registered. Participants were tasked with learning about fictitious minerals, which were greyscale rectangles (https://osf.io/sa6fq). The critical manipulation for Domain was implemented via the cover story, as well as the wording of the questions prompting responses in the training and generalization test phases (see Figure 1A and 1B). For Predictive Learning, participants were told that the stimuli were minerals that they were to process in a lab. Some of the minerals would lead to a fictitious outcome “Agog” (A), while others would lead to “No Agog” (noA), or “Bugi” (B), depending on condition. The cover story for Category learning involved classifying the minerals into different compounds. Participants were told that some of the minerals would be classified as “Agog”, and others would be classified as “No Agog”/“Bugi”. All other aspects of the presentation of the task (e.g., stimuli, feedback, timing etc.) were equated between domains.

After providing informed consent, participants answered basic demographic questions (gender, age, language) and were asked to adjust their screen (e.g., brightness) until two presented stimuli were distinguishable (the two center stimuli in Figure 1B). A brief instruction check quiz was presented to ensure that participants understood their task. If participants answered any questions incorrectly, they were presented with the instructions again. Once participants answered the questions correctly, they commenced the training phase.

#### Training.

During training, participants learned to respond to two visual stimuli with one of two outcome/category labels via trial-and-error feedback about the correct outcome (e.g., stimulus 3 → label A and 6 → label B). Each stimulus was only associated with one of the two labels, meaning that feedback was deterministic. There were 12 presentations of each stimulus. The order of presentation was randomized in 4 blocks of 3 presentations of each stimulus. Participants were either assigned stimulus 3 (lighter grey) to be associated with A and stimulus 6 (darker grey) to be associated with noA/B, or vice versa.

On each trial, participants were presented with S3 or S6, and either asked “What outcome do you predict will happen?” (Predictive Learning) or “What category does this mineral belong to?” (Category Learning). Participants made a forced-choice response by clicking on buttons labeled with Agog or No Agog/Bugi, which were presented below the stimulus. Feedback was presented containing information about the correct outcome, and participants clicked “Continue” once they were ready to proceed to the next trial. Note that this meant that the feedback duration was variable between trials and participants.

#### Test.

The test phase started immediately after the training phase after some instructions. The test phase consisted of multiple generalization tests presented in separate test blocks. To assess generalization, 8 stimuli along the whole range of the dimension were presented in randomized order (two trained and six novel, see Figure 1B). The total number of trials, wording of the test question (see Table 1), and response format depended on condition. For continuous probability ratings, participants saw each stimulus once and responded by clicking on an 11-point scale (0–10). For binary decisions, participants clicked on one of two response buttons and each stimulus was presented 10 times, to equate the granularity of the measure with the probability ratings. This made it possible for us to observe identical gradients between tests.

**Table 1.** Test questions and response options for each condition in Experiment 1.

**Format**	**Framing**	**Prediction**	**Categorization**
Probability	Absolute	Q. What is the likelihood of this object leading to Agog? [Agog 100% unlikely, Agog 100% likely]	Q. What is the likelihood of this object belonging to the Agog category? [Agog 100% unlikely, Agog 100% likely]
Probability	Relative	Q. What is most likely in terms of what this object will lead to? [No Agog/Bugi 100% likely, Agog 100% likely]	Q. What is most likely in terms of what category this object belongs to? [No Agog/Bugi 100% likely, Agog 100% likely]
Decisions	Absolute	Q. Will this object lead to Agog? [No, Yes]	Q. Does this object belong to the Agog category? [No, Yes]
Decisions	Relative	Q. Will this object lead to Agog or No Agog/Bugi? [No Agog, Agog]	Q. Does this object belong to the Agog or No Agog/Bugi category? [No Agog/Bugi, Agog]

*Note*. Response options or scale limits are in square brackets. Experiment 1 included factorial combinations of format and framing, Experiment 2 only included Absolute Probability ratings.

In Experiment 1, there were 3 test phases in total. Participants were tested separately on Framing (Absolute and Relative, between-subjects) but on both Formats (Decisions and Ratings, within-subjects in counterbalanced order). In the third and final test phase, we asked participants to provide Probability ratings for the alternative outcome (NoA in Predictive Learning and B for Category Learning). In Experiment 2, the order of the two tests (A and noA was counterbalanced i.e., Target was within-subjects), and we used a single format (Absolute Ratings). This meant that all factors in Experiment 2 were between-subjects manipulations except for the two outcome ratings (predictions for A and noA), which were presented in separate test phases in counterbalanced order (see Figure 1C). There was no feedback presented for any of the test phases in either experiment (Lee et al., 2022).

### GSM: Gaussian-Sigmoid Model

To analyze the generalization data, we developed a novel hierarchical Bayesian model which estimates descriptive features of individual and group-level gradients. A significant innovation of our approach is the use of a Markov-chain Monte Carlo (MCMC) model selection procedure (Lee & Wagenmakers, 2014; Sisson, 2005) to determine whether each individual’s gradient is best described by a single sigmoid or a combination of sigmoid and Gaussian components, akin to splined regression (Marsh & Cormier, 2002) and an extension of the Augmented Gaussian model proposed by Lee, Mills, et al. (2021). This enables us to capture both Gaussian and sigmoidal response patterns as a qualitative mixture of sub-populations (*p*[Gaussian] parameter), and to estimate quantitative parameters including the slope (gradient steepness: steeper gradients indicate more determinism/binary responding for ambiguous stimuli located between the training stimuli) and midpoint (location on the dimension where responding is at 50%: higher midpoints indicate a shift in the gradient or overall response bias towards one of the outcomes).

For each participant, both Sigmoid (*s*_*cki*_) and Gaussian (*g*_*cki*_) predictions were computed across stimuli, with model selection performed at each iteration in the MCMC method. This method determines whether the data are better described by a purely sigmoidal pattern (*z*_*ck*_ = 1) or a combination of a sigmoid augmented with a Gaussian (similar to Lee, Mills, et al., 2021) on the left side starting at an estimated transition point *γ*_*ck*_ (the target location near the trained stimulus of the queried outcome). This also serves to de-bias quantitative parameter estimates (e.g. slopes) by accommodating data that do not perfectly fit either assumed gradient shape alone (similar to ‘lapse’ cases in psychometric modeling; see Lee & Wagenmakers, 2014). For easier interpretation of effect size, the stimulus values were standardized (mean 0, *SD* 1), and *γ*_*ck*_ was constrained to be at or above stimulus 3 to avoid unidentifiable Gaussian slopes at the end of the stimulus dimension. To ensure continuity, the Gaussian predictions were scaled by the minimum Sigmoid value at the transition, aligning peak heights between functions.

In summary, while our model is a measurement model, it captures the fact that some individuals generalize using relational rules (yielding sigmoidal gradients), while others rely on similarity to the previously trained stimulus of the queried (target) outcome (producing Gaussian-like gradients) (see Figure 1D; see Lee et al., 2018; Lovibond et al., 2020; Rouder et al., 2004; Rouder & Ratcliff, 2006). It estimates the proportion of Gaussian-like gradients, and includes parameters capturing key features of generalization within and across the shape subgroups (shared sigmoid slope and midpoint, but unique additional downward slope for Gaussian subgroup). This means that our model captures multiple ways in which experimental manipulations might affect generalization. For example, asking participants to make binary decisions (rather than continuous ratings) at test may make generalization gradients more deterministic (increase slope), more rule-like (decrease *p*(Gaussian)), but have no effect on the midpoint.

We applied these models to the data using a Beta link function, and analyzed the effects of our manipulations using a contrast coding scheme with all main effects and interactions, and by-participant random intercepts. Hierarchical error terms were used to model participant-level variability. A logistic regression model matrix included all main effects and interactions of the central design factors, with contrast coding. Bayes Factors (BFs) were calculated via the Savage-Dickey density ratio (Dickey, 1971) relative to a zero effect (±0.01 density interval), and we interpreted the Bayes Factors using the guidelines suggested by Jeffreys (1961).

In Experiment 2, we deviated from our pre-registered approach by introducing an additional qualitative parameter, *p*(negation), which captures whether the noA gradient should be modelled as the negated A gradient (e.g., Gaussian around A, then inverted) or as independent from the A gradient (explained further below). In Experiment 2 we also implemented the additional constraint that if a participant was assigned to the Negation model (i.e., A ratings were treated as inverted NoA Ratings), we applied the exact same slope and midpoint parameters to both A and NoA gradients, otherwise, these estimates were treated as independent. Full model specification is provided in the Supplemental Materials.

## RESULTS

### Experiment 1

**Figure 2.**
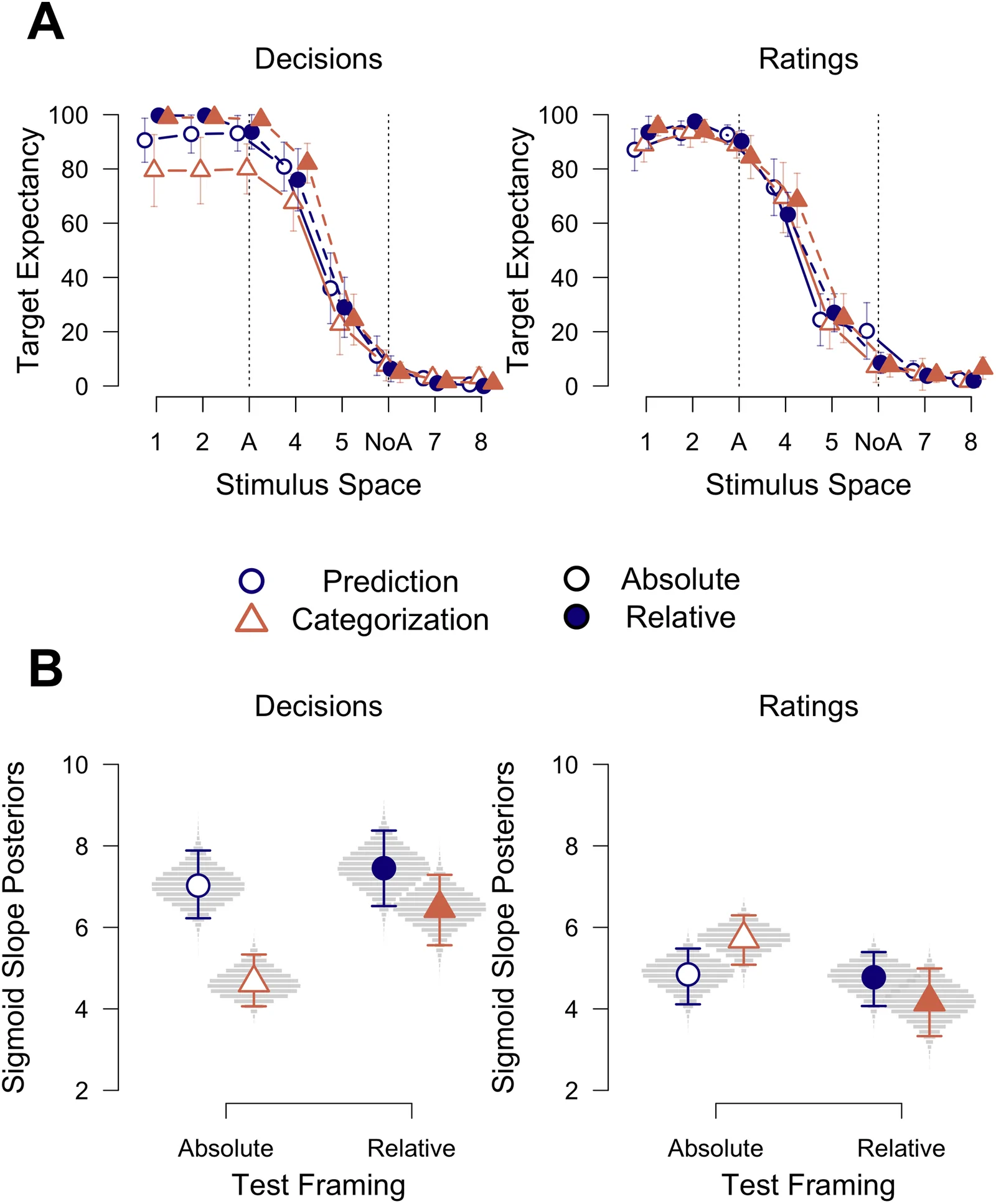
(A) Generalization gradients for each condition in Experiment 1 (*N* = 277). (B) Posteriors for the slope parameter for each condition in Experiment 1.

Experiment 1 manipulated both Format and Framing between Predictive and Category learning domains (see Figure 1B and Table 1 in the Methods for the exact test questions) and included a second set of test phases counterbalancing the test Format within participants. Due to the presence of order effects (flatter gradients for Ratings when tested after Decisions, see Supplemental Materials^2^), we decided to analyze the first test only, making Format a between-subjects factor. Note that the central results regarding the preregistered main effects of Format and Framing in the first test phase replicated in the second test phase (see Supplemental Materials), but not all their interactions. Figure 2A shows the generalization gradients for each condition and Table 2 shows the computed Bayes Factors (BFs) for each effect in Experiment 1.

**Table 2.** Bayes Factors for effects for each parameter.

**Effect**	**Qualitative Parameters**		**Quantitative Parameters**	
	***p*(Gaussian)**	***p*(Negation)**	**slope**	**midpoint**
Experiment 1				
Domain	0.356		6.50	0.468
Format	1.53		>1,000	0.028
Framing	>1,000		0.161	0.033
Domain × Format	0.324		47.5	0.016
Domain × Framing	0.297		0.141	5.31
Format × Framing	0.622		42.7	0.009
Domain × Format × Framing	0.401		5.10	0.047
Experiment 2				
Domain	0.359	0.421	>1,000	0.992
Training	0.443	0.369	0.156	0.029
Target	146.7		>1,000	>1,000
Domain × Training	0.31	0.35	1.28	0.021
Domain × Target	112.2		>1,000	>1,000
Training × Target	0.33		0.038	0.022
Domain × Training × Target	0.336		0.979	>1,000

*Note*. List of factors: learning Domain (Categorization vs. Prediction), test Format (binary Decisions vs. continuous Ratings), test Framing (Relative vs. Absolute), outcome labels presented during Training (AvB vs. AvnoA), queried test Target (A vs. noA).

**Figure 3.**
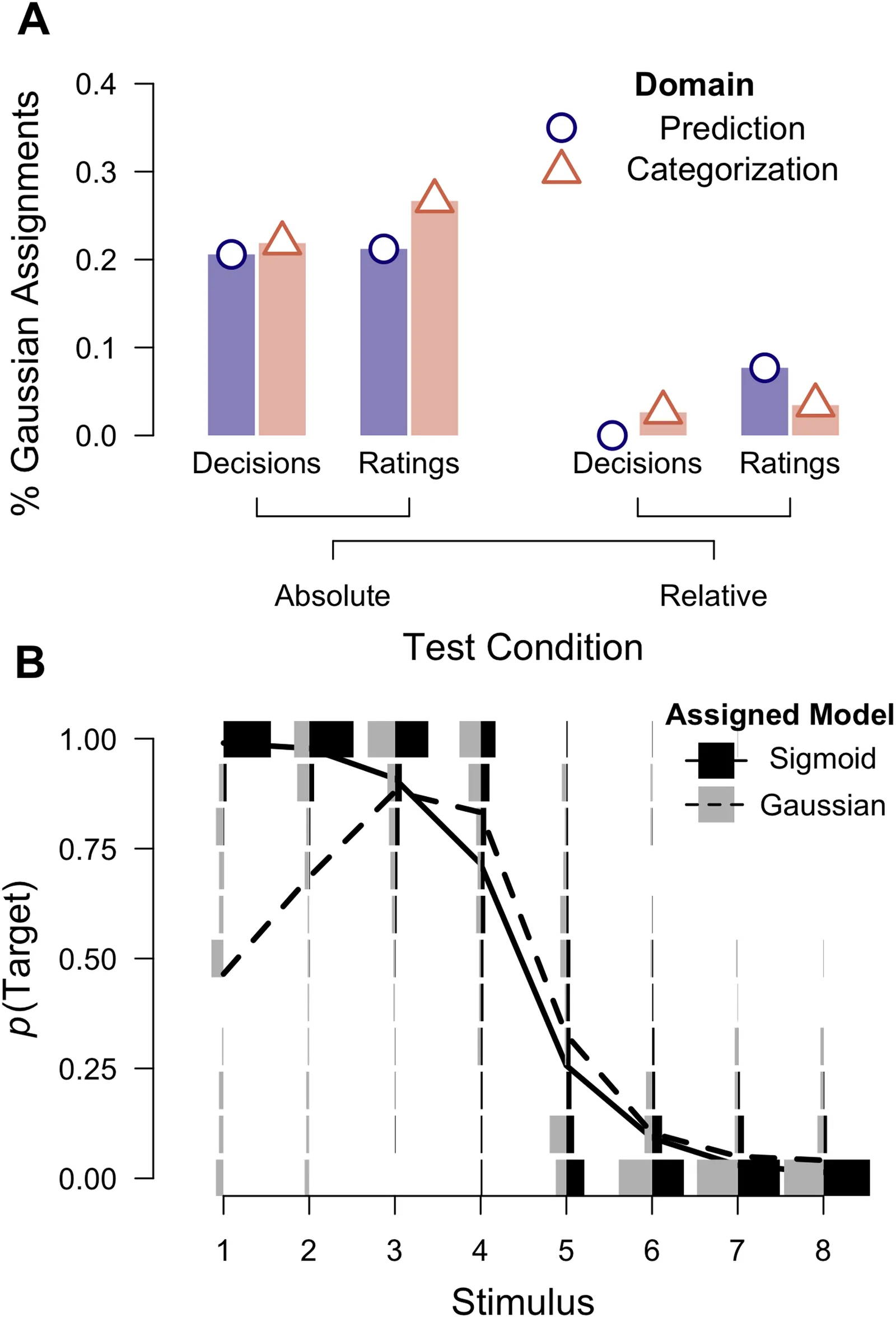
(A) Proportion of participants in each condition in Experiment 1 assigned to the Gaussian model with >80% confidence. (B) Generalization gradients for each assigned model in Experiment 1 (total *N* = 265 of *N* = 277, with *N* = 34 assigned to Gaussian). Histograms reflect distribution of responses.

As hypothesized, we found very strong evidence in support of a main effect of Format on the slope parameter (BF > 1,000) and a main effect of Framing on the *p*(Gaussian) parameter (BF > 1,000). Figure 2A shows that the effect of Format on slope is due to repeated binary Decisions producing more steep/deterministic gradients compared to probability Ratings. Regarding the shape parameter (Gaussian vs. sigmoid), Absolute test Framing led to more (∼20% difference) Gaussian-like gradients compared to Relative Framing, illustrated in Figure 3A (Absolute vs. Relative). All other main effects on the other parameters were in support of the null (BFs < 1/3) or anecdotal (BFs between 1/3 and 3).

In contrast to our hypotheses, we found moderate support for a main effect of Domain on the slope parameter (BF = 6.5), in that there were steeper gradients for Prediction than Categorization. This main effect was accompanied by an interaction between Domain and Format (BF = 47.5) and a further three-way interaction with Framing (BF = 5.1). A visual inspection of the posteriors in Figure 2B reveals that the slope estimates for Prediction do not differ between Absolute and Relative framing, but they do for Categorization. One interpretation of these results is that participants are not encoding the alternative outcome in Prediction, the absence of A (noA). Thus, when participants are tasked with integrating their learning about noA to make relative judgments, there is no difference between their Absolute and Relative judgments because there is nothing to integrate. In Categorization, participants learn about both categories (A and B) in training and thus Framing does make a difference. It is not clear why the pattern of results differs between Decisions and Ratings but it is worth noting that this odd and unexpected pattern of results did not replicate in the subsequent test phase where we counterbalanced the Format within participants, and thus we are hesitant to interpret these interactions further (see Supplemental Materials).^3^

In summary, Experiment 1 found strong evidence that Format and Framing affected different descriptive features of generalization gradients, with Format affecting the gradient slope and Framing affecting gradient shape. Thus, it seems that Framing affects response rules (more rule-based responding with relative framing, more similarity-based generalization with absolute framing), while Format affects response determinism (more binary responding with Decisions than Ratings). We also found some interactions with learning Domain, but these did not replicate in the second test phase and we are therefore hesitant to interpret these results. Another reason to be cautious is because in choosing to implement the most commonly used designs for Predictive and Category learning, Experiment 1 confounds Domain with the outcomes presented to participants during training. In Prediction, participants learned about a single outcome that was present or absent but in Categorization, participants learned about two different outcomes. Given this confound, it is not clear whether differences between Domains reflect differences in learning about categories and outcomes, or differences in learning about the *alternative* outcome, which was an absent event (noA) in Prediction but a different event (B) in Categorization. Since the effects of the other procedural manipulations were relatively clear, in Experiment 2 we focused on disentangling these two factors, and isolating the role of the alternative outcome (noA or B).

### Experiment 2

The aim of Experiment 2 was to test whether generalization is affected by learning Domain (Prediction vs. Categorization) or by the labels of the observed outcomes in Training (AvB or AvnoA; see Figure 1C). For simplicity we decided to compare generalization gradients using a single test format (Ratings with Absolute framing). Based on the results of Experiment 1, we chose to use Ratings to minimize potential ceiling effects (deterministic slopes) and floor effects (no Gaussian shapes) that might be obtained if we used Decisions. Unlike Experiment 1, we asked for judgements about outcome A, as well as the alternative outcome noA, in counterbalanced order^4^. We chose to ask about noA rather than B to equate the testing procedures between training conditions (AvnoA and AvB).

**Figure 4.**
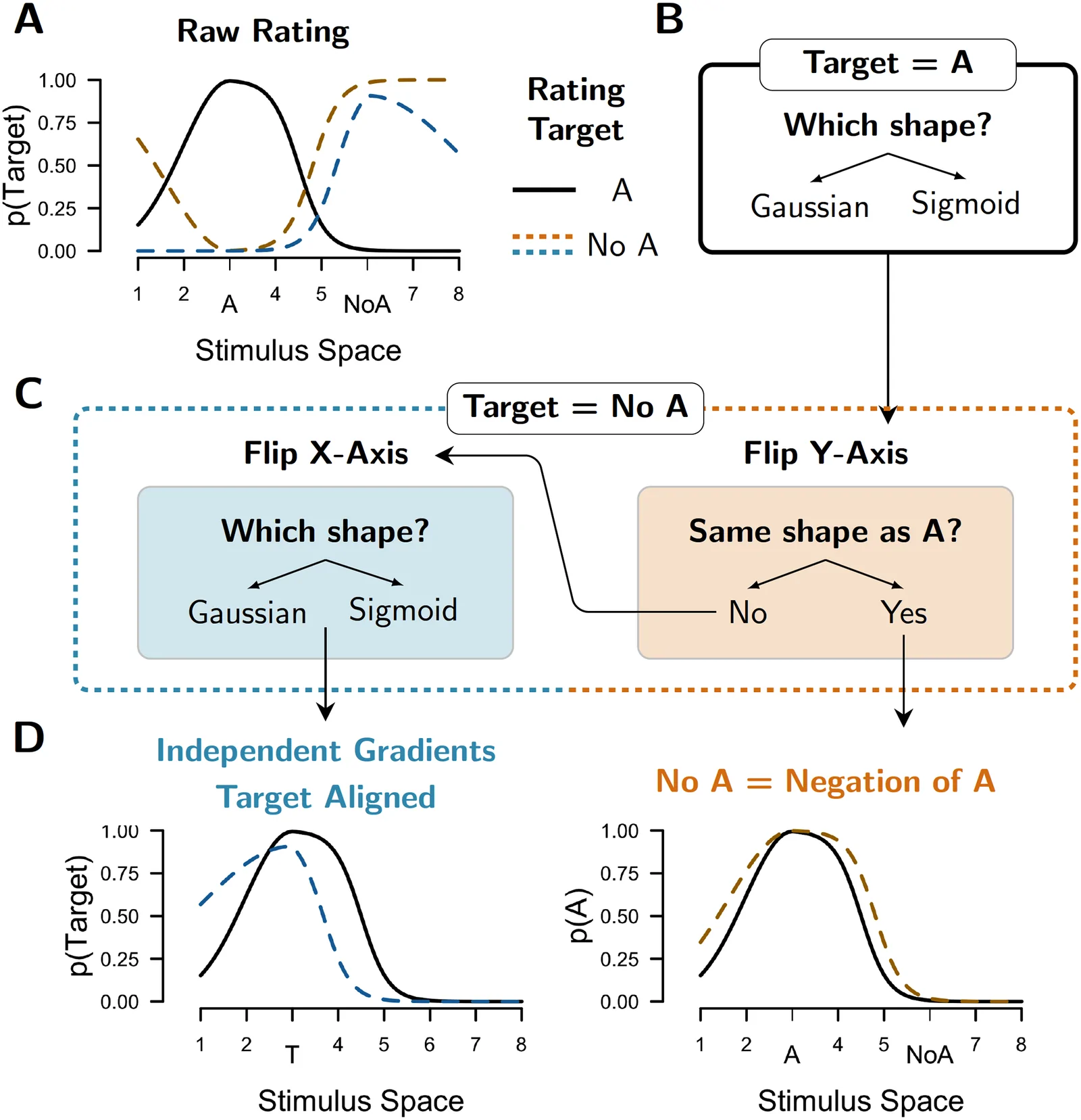
Depiction of model selection procedure in Experiment 2 with qualitative parameters capturing individual differences in A gradients (Gaussian vs. Sigmoid) and noA gradients (negated vs. independent). (A) Example of raw A and two potential NoA gradients. Note that the peak location is not aligned and thus the gradients are not comparable without transformation. (B) *p*(Gaussian) for A ratings determined by classifying gradients as Gaussian or Sigmoid (C) No-A ratings were then inverted (flipped over *y*-axis), and compared to A gradient. If they were the same shape, the noA gradient was classified as negated. If not, the noA gradient was classified as independent, and further classified as Gaussian or Sigmoid, similar to the A gradient (D) Example of how noA gradients were aligned to facilitate comparison with A gradients if they were independent (blue gradient, flipped over the *x*-axis, left panel) or negated (orange gradient, inverted over the *y*-axis, right panel).

Experiment 2 required a modification to our measurement model. The A and noA gradients peak at different locations on the dimension and therefore require transformation in order to be compared. Our pre-registered approach was to align the gradients in terms of their target location by flipping the noA gradients over the *x*-axis. However, examination of individual gradients revealed that a subset of the gradients for the noA outcome appeared similar to the y-inverted, instead of x-inverted, gradient for the A outcome, suggesting that participants were learning about noA as the inverse or *negation* of A. We thus introduced a *p*(negation) parameter in our Bayesian model, which determined whether the noA gradient was modelled as the inverse of the A gradient, or independent from the A gradient. Figure 4 shows that this model selection procedure involves first estimating the gradient shape (sigmoid or Gaussian) for the A gradient, similar to the method in Experiment 1. Thereafter, we evaluate which transformation of the noA gradient (flipping over *y*-axis or *x*-axis) better corresponds to the A ratings. If the y-flipped shape is similar to the A gradient, then we classify the noA gradient as negation and flip it over the *y*-axis, otherwise, it is classified as independent and instead flipped over the x-axis (see Figure 4D) for analysis. The analysis was otherwise identical to Experiment 1. We calculated BFs for main effects and interactions involving Domain, Training outcomes, and test Target factors^5^.

**Figure 5.**
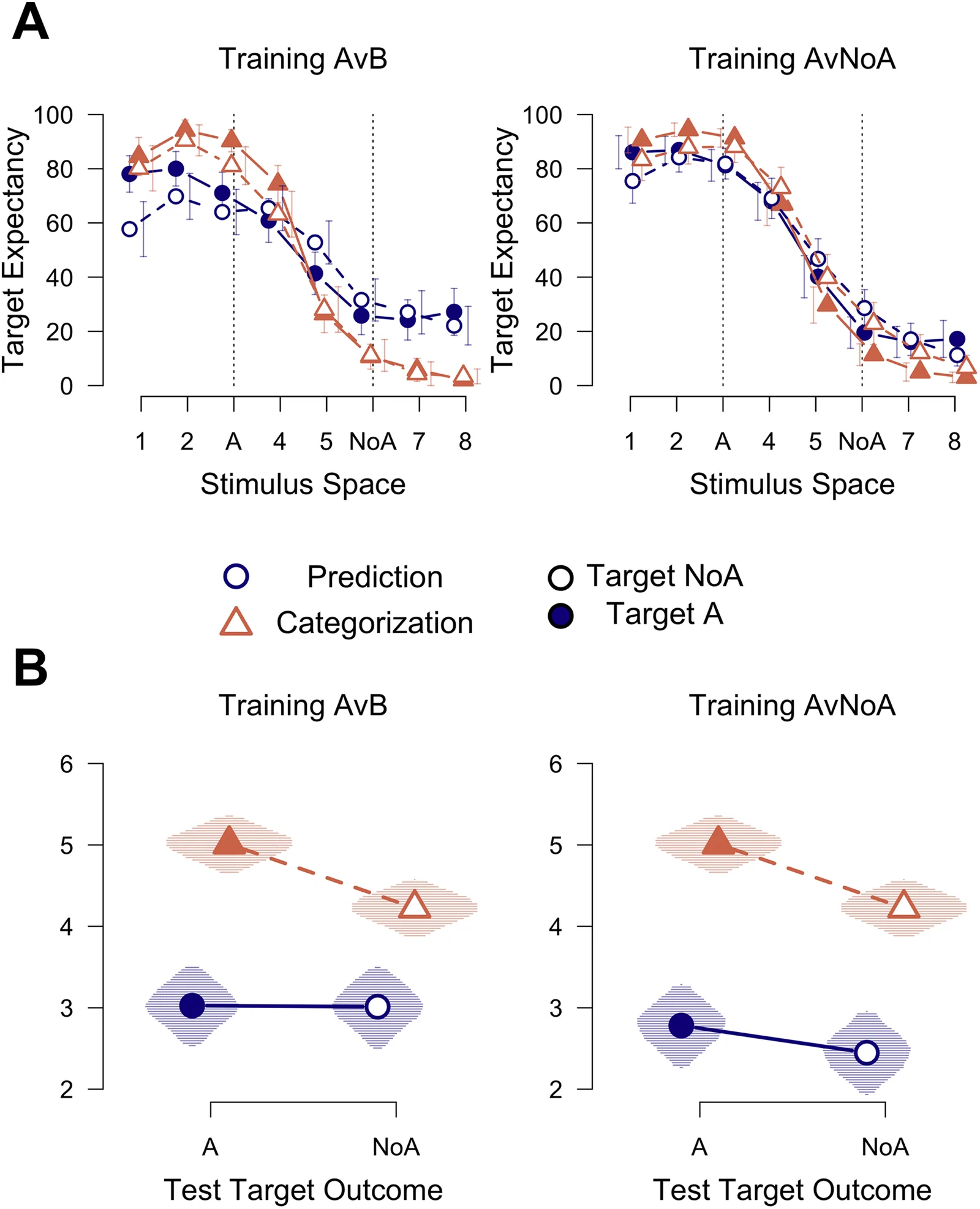
(A) Generalization gradients in Experiment 2 (*N* = 245) for AvB (left) and AvnoA training conditions (right). (B) Posteriors for the slope parameter for each training condition in Experiment 2.

Figure 5A shows the gradients for each condition and Table 2 shows the Bayes Factors for each effect in Experiment 2. In terms of main effects, the only effect of Domain was found for the slope parameter (BF > 1,000), with steeper gradients in Categorization than Prediction (replicating the domain effect in the Absolute Ratings design cell in Experiment 1). A straightforward interpretation of this effect is that when asked to categorize, participants become more categorical (i.e., deterministic) in their ratings. It should be noted that Experiment 1 demonstrates the opposite pattern when participants made binary decisions, with steeper slopes in Prediction than in Categorization (see Figure 2B). This somewhat bizarre pattern of results might be explained by assuming different default modes of learning in Prediction and Categorization. Categorization is inherently binary, and thus asking participants to make Decisions at test is natural and does not make categorization any more deterministic whereas this does occur for Prediction. Even though we asked participants to give absolute judgments for a single outcome in Experiment 2, Categorization participants may have still imposed a relative framing on the judgment, leading to steeper gradients in Categorization than Prediction. An alternative proposal is that all participants attempted to make absolute judgments, but it is more difficult to disentangle categories than outcomes (a potential reason is discussed below).

**Figure 6.**
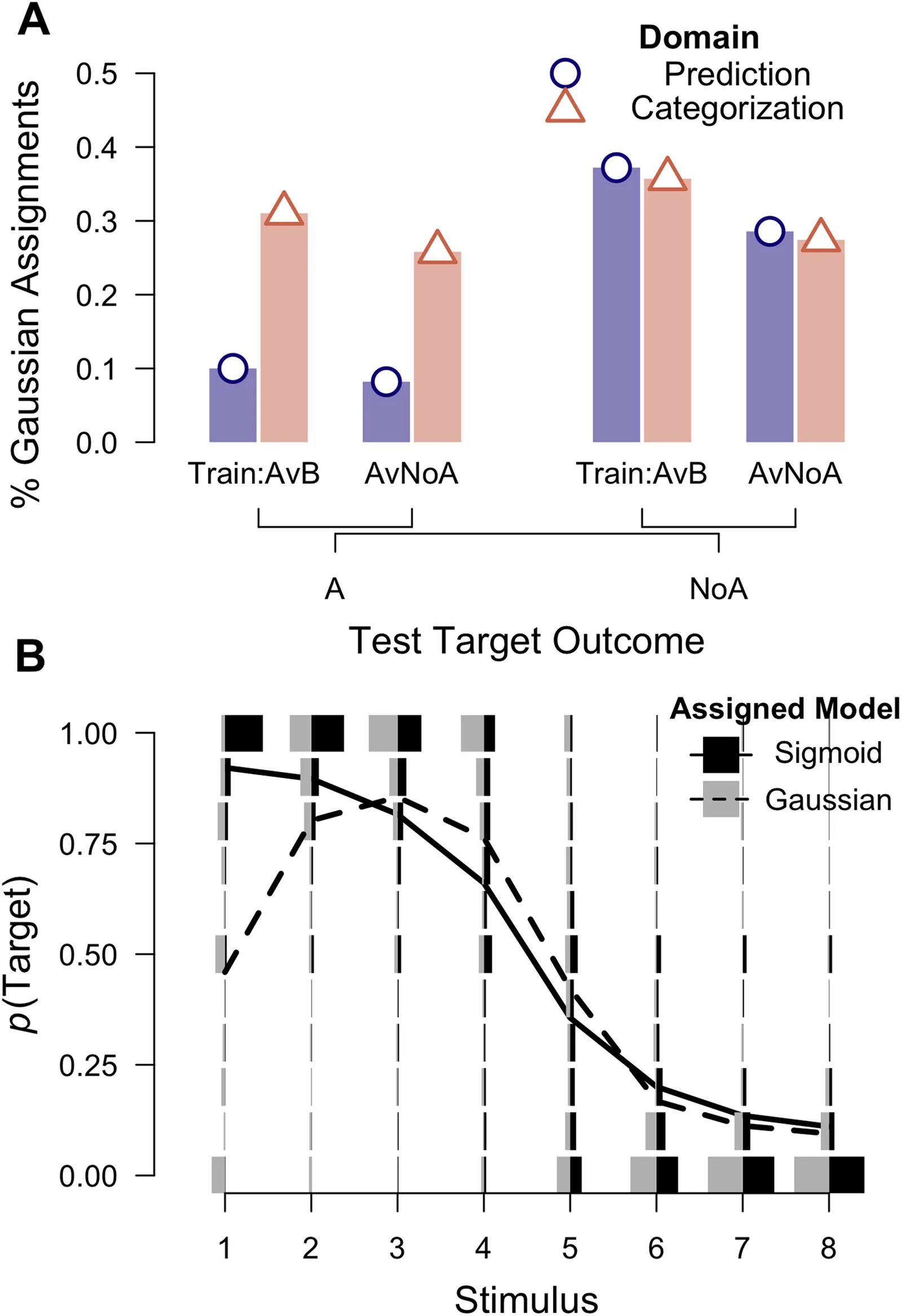
(A) Proportion of participants in each condition in Experiment 2 assigned to the Gaussian model with >80% confidence. (B) Generalization gradients for each assigned model in Experiment 2 (total *N* = 265 of *N* = 277, with *N* = 34 assigned to Gaussian). Histograms reflect distribution of responses.

The most important result from Experiment 2 was an interaction between Domain and Target which occurred for the *p*(Gaussian) (BF = 112.2), slope (BF > 1,000), and midpoint (BF > 1,000) parameters. Examining Figure 5A and 6A, this two-way interaction appears to be driven by the fact that gradients for A and noA are more different for Predictive (less steep, shifted, and Gaussian for noA) than for Category learning. To interpret these results, imagine if participants learned about both outcomes/categories in a perfectly mutually exclusive way. This would mean that if a stimulus led/belonged to A, it would not lead/belong to the alternative (noA or B), and vice versa. If this were the case, we should expect to see identical A and noA gradients, since the outcomes would be treated as functionally equivalent. The similarity in the A and noA gradients for Categorization therefore suggests that participants treat the absence of a category (noA) as more or less equivalent to an alternative category B, but they do *not* equate the absence of an outcome (noA) to an alternative outcome (B). Interestingly, the A and noA gradients still differ for the AvnoA Training conditions, suggesting that people learn about absent events (noA) differently to present events (A), even when noA is presented explicitly as a possible outcome during training (see Figure 5A). Finally, the results for *p*(negation) were anecdotal for all effects (BFs <= 0.421), but it is worth noting that *p*(negation) was numerically higher for Categorization, consistent with the assumption that categories are represented as mutually exclusive and dependent, while outcomes are represented more independently.

We also found main effects of test Target, with strong evidence for an effect for *p*(Gaussian) (BF = 146.7), slope (BF > 1,000), and midpoint (BF > 100) parameters. Overall, the gradients were more Gaussian and less steep, for noA gradients compared to A gradients. The *p*(Gaussian) results tell us that participants are less likely to integrate noA with the non-queried outcome (A) in a relational rule. Instead, participants made absolute ratings about noA in a manner suggesting similarity-based generalization. Finally, the Bayes Factors provided anecdotal to moderate support for the Null for all parameters for the effect of Training labels (BFs <= 0.443), despite the gradients appearing to differ visually between AvB and AvnoA conditions (see Figure 5A).

## DISCUSSION

In this study, we compared generalization between predictive learning (learning predictive associations between stimuli and outcomes) and category learning (learning categorical associations between stimuli and categories), to investigate whether generalization exhibits universal characteristics as proposed by Shepard (1987). We tested this thesis by examining whether the gradients displayed across learning domains were of similar functional form, and by examining whether our experimental manipulations affected each domain in similar ways. Following Hendrickson et al. (2019), we made these comparisons while controlling task features that typically differ between learning domains.

Experiment 1 found that empirical differences between categorical and predictive generalization gradients can be largely explained by differences in testing procedure (the format and framing of the test judgments). Binary decisions produced more deterministic (steeper) gradients compared to continuous probability ratings, while judgments that involved a relative comparison of the likelihood of both outcomes produced more sigmoidal (and less Gaussian) gradients compared to absolute judgments involving a single outcome. In other words, test format influences response determinism (binary responding) while test framing influences response rules (similarity vs. rule-based responding). While it may seem fairly intuitive that judgments involving decisions produce more deterministic responding, the latter finding that relative versus absolute framing affects qualitative features of the gradient (whether a Gaussian or sigmoidal function is obtained) is theoretically significant since the shape of the gradient has often been used to understand the basis for generalization (e.g., rules vs. features/similarity, Lee et al., 2018; Livesey & McLaren, 2009; Lovibond et al., 2020; Wills & Mackintosh, 1998). The results we obtained suggest that this is not strictly true, or at least, that consideration of the testing method is needed to properly diagnose the content of learning (see also Gronau et al., 2023). This means that relational rules may not necessarily be derived during learning, but may be partly elicited by a testing procedure that promotes integration of both categories/outcomes. A related alternative is that both representations develop during learning but participants decide at test how to apply their knowledge (consistent with Lee & Livesey, 2018). In both cases, these framing effects suggest that one reason for individual differences in gradient shapes (Lee et al., 2018) is that some participants integrate learning about both outcomes to make their judgments (rule-based responding), while others consider the target outcome only (similarity-based responding, consistent with Wills et al., 2000). Our findings can also help to explain the differences between test formats observed in Hendrickson et al. (2019), since their forced-choice and probability ratings differed in both Format and Framing. In summary, the results from Experiment 1 explain why gradient shapes can differ between individuals (e.g., Lee et al., 2018) and between experiments or conditions with different testing procedures.

Experiment 2 manipulated a different factor which proved to be critical in uncovering differences between learning domains. In Experiment 2, participants either learned about a single outcome that was present or absent (A and no A) or about two outcomes (A and B). Here, our key finding was that A gradients were more sigmoidal (less Gaussian) relative to noA gradients, and this difference was larger for predictive learning than category learning. The larger differences between A and noA gradients in predictive learning suggest that participants do not equate the absence of the target outcome (noA) with the alternative outcome (B) in training. This makes sense given that outcomes occur in the world as independent events. What is perhaps surprising was that this also occurred for the AvnoA condition (we did not find a further interaction with Training condition) where noA was presented as an explicit outcome. In simplistic terms, participants’ judgments of the likelihood of A and the absence of A do not sum to 1 for each stimulus, suggesting a fundamental difference in the way that people learn about absent and present outcomes in predictive learning^6^.

### What Differs Between Predictive and Category Learning?

We believe that our observed differences between learning domains can be explained by considering two related ideas. The first is that categories are generally designed to be mutually exclusive (if something does not belong to A, it must belong to B), while outcomes are more independent. An alternative way of thinking about the distinction is that for outcomes A and B, there is an implicit third outcome that is possible (nothing: where no outcomes occur), but with category learning, participants do not spontaneously consider that a stimulus could belong to neither category. Thus, when participants were asked to make judgments about the absence of category A, they treated this judgment as equivalent to category B. This assumption of mutual exclusivity can explain why learning about the presence and absence of A was more similar for category than predictive learning across the slope, *p*(Gaussian), and midpoint parameters. It can also help explain why people became more deterministic in their judgments (slope effect for Domain) when asked to make categorical as opposed to predictive ratings, an effect which was observed in both experiments (but note that the opposite seems to occur for decisions). In other words, the reason that people become more categorical when making category judgments is because A and B (or A and no-A) are the only, mutually exclusive options. For prediction, it is less clear how the outcomes are related since outcomes occur independently in the world.

What is perhaps surprising is that we did not find interactions with training outcomes. It did not seem to matter whether the alternative outcome was B, or the absence of A. In particular, our results regarding the shape (*p*(Gaussian)) parameter are slightly perplexing. Experiment 2 showed that the proportion of Gaussian-like gradients was similar for A and noA gradients for category learning, but in predictive learning, noA gradients were much more Gaussian than A gradients (see Figure 5A). This means that in contrast to our intuitions, prediction gradients for A were more sigmoidal than categorization gradients for A. At face value, this result seems to contradict our domain effects for the slope parameter. If we assume that categorization makes people more deterministic/binary, we should predict the opposite result (more sigmoidal gradients for categorization than prediction). Therefore to explain this pattern of results, we need to consider another idea.

The differences in gradient shape might be explained if we assume there is a fundamental difference in how absent events are conceptualized between domains. In categorization, the absence of A (noA) is a defined alternative and therefore treated as equivalent to B, making absent categories a concrete, mutually exclusive counterpart to present categories. Thus, asking participants to make judgments about A and noA at test engage the same tendency to contrast the categories (A vs. B/noA) as they are viewed as opposites, yielding similar mixtures of Gaussians and sigmoids for A and noA. In prediction however, noA is an ambiguous, residual collection of events encompassing all non-A outcomes (e.g., other outcomes occur, nothing occurs) since as stated above, outcomes occur independently and are not necessarily mutually exclusive. This may mean that when participants are queried about their predictions for A, they contrast A against this heterogeneous collection of noA outcomes, producing more rule-based (sigmoidal) gradients. But when directly queried to make predictions for noA, they lack a coherent outcome to contrast since noA can encompass multiple combinations of events, and thus participants default to similarity-based generalization (producing more Gaussian gradients). In other words, there is an asymmetry in representation of present and absent events in predictive learning. This explanation implies that in prediction, participants treat absent outcomes as an independent construct that can be associated with stimuli, and not merely the negation of a present outcome as in categorization. Note that although the BF was anecdotal, our results regarding the *p*(negation) parameter were consistent with this interpretation (more negation for categorization than prediction).

Notably, our cover story did not inform participants about the relationship between the categories or outcomes to each other. Participants seem to have applied their assumptions to the task, and generalized accordingly. Since our manipulations were purely based on verbal instructions, our results are important in suggesting language (e.g., labeling consequences as categories) as a moderator of outcome representations that affect learning. There may also be other subtle differences between domains. Due to the temporal factor involved in prediction (i.e., the requirement to make predictions for the future based on the past), it may be that predictive judgments involve more uncertainty and are therefore made less confidently than categorical judgments (stimuli that belong to categories in the past tend to belong to the same category in the future). Differences in confidence may explain why the gradients for prediction are flatter than those for categorization (see Figure 5A). Future research can test these idea experimentally, as well as examine whether existing models of learning can accommodate our findings.

One existing model that may be compatible with our findings is Schlegelmilch et al. (2022)’s Category Abstraction Learning (CAL) model. This model contains a “contrasting” parameter, which assumes that similarity-based generalization occurs not just for outcomes that are present at a given moment, but also for other outcomes that are not present (via dissimilarity to the present stimulus, Stewart & Brown, 2005). Such a process instantiates the idea that the presence of an outcome for a current stimulus implies the presence of a different outcome for dissimilar stimuli. The assumption of mutual exclusivity could be implemented in the model via varying levels of contrasting, either as a function of domain, or as a function of test targets. Schlegelmilch et al. (2022) simulated a typical predictive learning task by assuming a flatter gradient for NoA generalization, which may help to account for the effects observed here as well. However, the interactions we found in Experiment 1 (e.g., Domain and test Format) are non-trivial to model since they involve not just learning but interactions with processes that read out learned information at test (e.g., choice rule selection), highlighting the importance of capturing processes involved in learning and testing in future modeling work.

### Is Generalization General?

In light of our results, the answer to our original question of whether generalization exhibits universal characteristics is not straightforward. Our first test was to examine whether the functional form of generalization differed between predictive and category learning while controlling for procedural differences between tasks. Our second test was to examine whether our experimental manipulations (format, framing, training outcomes) affected generalization in each domain in the same way, similar to Hendrickson et al. (2019). For both tests, we found similarities in generalization between domains, suggesting that generalization does exhibit universal characteristics as proposed by Shepard (1987) and that both tasks tap into the same underlying process. Critically, unlike Hendrickson et al. (2019), we obtained null results for qualitative differences in gradient shape across different response formats, suggesting that predictive and category learning involve the same mixture of similarity- and rule-based processes. Gradient shape was however, affected by methodological manipulations (relative vs. absolute framing), a study feature that often varies between categorization (relative), and prediction (absolute). These results highlight the importance of de-confounding procedural differences in order to understand underlying processes in different tasks.

The clearest differences between domains we obtained were steeper gradients in category learning than predictive learning (when using continuous ratings), and an interaction with test target (A vs. noA) with greater similarity between A and noA gradients in category learning than predictive learning. As stated above, we believe that both of these results can be explained by greater mutual exclusivity of categories compared to outcomes, and differences in conceptualization of absent categories vs. outcomes.

Although we did not obtain the expected set of null results, in our view, the differences that we observed do not contradict Shepard (1987), but rather, complement them. Shepard explained observed invariances in behavior through a process of internalizing properties of “natural kinds” through the course of evolution. Here, we add to this view by proposing that differences in generalization of category learning arise through internalization of knowledge about what we term *cognitive kinds*. Categories are created by us for a specific purpose. We understand that the function of categories is to sort stimuli into one of many options, and thus our behavior reflects this assumption of mutual exclusivity of categories in a way that does not apply for outcomes (see also Goldstone et al., 1996). In this way, cognitive kinds can be seen as similar to artifacts (objects created by humans for a specific purpose, e.g., chair) in that both are defined by human intent, and are therefore reasoned about differently than natural kinds (Bloom, 1996; Keil, 1992; Malt & Sloman, 2007). This explains why we do not generalize learning about categories in the same way as predictive associations about consequential outcomes, which are more biologically primitive. This interpretation implies that the mechanisms underlying predictive and category learning are the same, with differences confined to how categories/outcomes are represented in relation to each other. Thus, we extend on Shepard’s thesis in claiming that empirical differences in generalization gradients can be explained by differences in stimulus representation (i.e., distance in psychological space as proposed by Shepard, 1987), as well as by differences in *outcome* representation (mutually exclusive or independent). The intriguing implication of this statement is that if these implicit assumptions about categories and outcomes could be equated, then our observed differences may disappear, which would warrant a more integrated view of predictive and category learning.

### Limitations

There are some limitations of our study worth noting. First, unlike Shepard (1987), we did not rescale our gradients according to psychological similarity, and instead analyzed the raw gradients. We therefore cannot conclude whether differences in psychological space between predictive and category learning might instead explain our results. While we did not find key differences in gradient shape between domains, it remains to be seen whether the domain differences disappear once the gradients are rescaled according to other distance metrics. Another limitation of our study is that unlike typical category learning studies, our study contained a single exemplar from each category. This decision was necessary to keep the design as simple as possible in order to isolate the learned associations between stimuli and outcomes. It is possible that with multiple exemplars, other assumptions may come into play. For example, participants may assume that stimuli that lead to the same outcome are less similar than stimuli that belong to the same category (related to the acquired equivalence effect, Honey & Hall, 1989). Our conclusions are also limited to instances in which categories are learned via a discrimination (i.e., classification) task. Previous research shows that alternative ways of learning about categories (such as feature prediction tasks) lead to differences in category representations (Chin-Parker & Ross, 2002; Goldstone, 1996; Markman & Ross, 2003), which is an interesting avenue for future research to explore.

It is important to highlight that although we detected some differences in the mixtures of Gaussian and sigmoid gradients, overall, the gradients were predominantly sigmoidal in shape. This was particularly surprising for the predictive learning conditions, since previous work has shown an approximately equal mixture of rule- and similarity-based gradients (e.g., Lee et al., 2018). One explanation for this result is that we used a test dimension (light/dark) with clearly defined endpoints that are self-evidently opposing (black and white). This contrast may have encouraged more binary responding (i.e., rule learning) compared to continuous dimensions with less defined endpoints (e.g., green-blue hue, circle size), and may have also created some ceiling effects in our data (e.g., relative framing conditions in Experiment 1). Replication with other stimulus dimensions is therefore needed to test the robustness of our results concerning gradient shape. In any case, our results highlight the importance of accounting for qualitative differences in gradient shape when analyzing generalization gradients.

### Conclusion

In summary, using a carefully controlled task design, we found similarities, but also meaningful differences in generalization gradients between predictive and category learning domains. Differences in generalization gradients can largely be explained by procedural factors in testing (Experiment 1) and assumptions that participants make regarding the mutual exclusivity of categories and outcomes and representation of absent events between domains (Experiment 2). Our results underscore the importance of controlling for procedural factors when interpreting and modeling generalization gradients. Given that the majority of our results demonstrated similarities between domains, we conclude that predictive and category learning appear to be instances of the same basic phenomenon (associative learning), and both tasks assess the same fundamental process of generalization. We propose that the observed differences in generalization between domains reflect internalized assumptions about the way in which categories and outcomes operate in the world, namely, that categories are represented in a more mutually exclusive way than outcomes. In all other respects, generalization does appear to be general across predictive and category learning domains.

## FUNDING INFORMATION

J.C.L. was funded by a Discovery Early Career Researcher Award from the Australian Research Council (DE210100292).

## AUTHOR CONTRIBUTIONS

J.C.L.: Conceptualization; Writing – original draft; Writing – review & editing. A.J.W.: Conceptualization; Writing – review & editing. R.S.: Conceptualization; Formal analysis; Methodology; Software; Writing – original draft; Writing – review & editing.

## DATA AVAILABILITY STATEMENT

Data and analyses are publicly available on OSF (https://osf.io/9jzpr).

## Notes

^1^ Here we refer to the type of problem facing the organism, not the class of theoretical processes described as “associative learning” (see Wills, 2005).^2^ In the Supplemental Materials we highlight how the two measures of ratings and decisions change in their sensitivity for choice behavior, following up on previous research (Lovibond et al., 2020).^3^ The main effects of Format (slope) and Framing (shape) did replicate in test phase 2. The Domain × Framing effect regarding the midpoint parameter (BF = 5.31) also did not replicate in test phase 2, and therefore we do not interpret this effect (see Supplemental Materials).^4^ We also measured noA/B ratings in an exploratory test phase in Experiment 1, see Supplemental Materials.^5^ We also included test order as a factor but we omit these results for brevity and because the effects were not substantial (see Supplemental Materials for full results).^6^ Presumably, this asymmetry is stronger for conditioning experiments involving non-hypothetical outcomes.

## Supplementary Material


