# Supplementary material for: Is Generalization General? Differences Between Predictive and Category Learning Reflect Knowledge of “Cognitive Kinds”
Source: Open Mind (Camb). 2026 Jul 17;10:1017–38. doi: 10.1162/OPMI.a.368 (PMC13518074; doi:10.1162/OPMI.a.368)
Supplement: Supplementary file 1 [file opmi-10-1017-s001.pdf]

Supplemental Material: Is generalization general? Differences between predictive and category learning reflect knowledge of "cognitive kinds"

Jessica C. Lee[1,2], Andy J. Wills[3], and René Schlegelmilch[4]

[1]University of Sydney; [2]University of New South Wales; [3]University of Plymouth; [4]University of Bremen

Correspondence concerning this article should be addressed to René Schlegelmilch.

University of Bremen, COGNIUM

Hochschulring 18 / R0430, 28359 Bremen, Germany

E-mail: r.schlegelmilch@uni-bremen.de

Supplemental Material: Is generalization general? Differences between predictive and category learning reflect knowledge of "cognitive kinds"

Bayesian Model

The Bayesian model (Figure 1) was inspired by the augmented Gaussian model by J. C. Lee et al. (2021) designed to capture rule-like (sigmoidal) and similarity-like (Gaussian) gradient patterns. Lee et al.'s original goal was to quantify and test observed effects such as the peak shift phenomenon (Hanson, 1959) in predictive learning tasks, for which there was no adequate statistical modeling solution. The original model by J. C. Lee et al. (2021) combines two Gaussian functions with separate width parameters to capture the descent on the left and right of the estimated peak-location in the stimulus space (i.e., around the CS+ [conditioned stimulus predicting the outcome]). However, the model was constrained to capture rule- and similarity-like gradients via this peak location parameter, and therefore did not fully capture individual differences in gradient shape in the manner that we required. Since one of our goals was to test whether our experimental manipulations affected the prevalence of these sub-populations (similarity and rule-based generalization), we took this mixture of populations into account by implementing a Bayesian model selection with hierarchical variance terms within each subgroup.

Specifically, we implemented a mixture of Sigmoid and Gaussian gradients (see graphical model in Figure 1 and bottom row of Figure 2). First, for each individual k in condition c, both Sigmoid and Gaussian predictions are calculated over the stimuli i (i.e., s_{cki} and g_{cki}, respectively). These predictions depend on the priors shown in Figure 1 and are explained further below. The model selection is based on a transdimensional Markov Chain Monte-Carlo (MCMC) method (M. D. Lee & Wagenmakers, 2014; Sisson, 2005), which in each iteration, estimates whether the gradient is purely sigmoidal ($z_{ck} = 1$) or should be augmented (spliced) with a Gaussian ($z_{ck} = 2$) at the estimated peak location γ_{ck} on the left-hand side (p[Gaussian], see Figure 2). The term 'left-hand' thus refers to the notation in the graphical model (Figure 1), stating 'if $z_{ck} = 2$ for $x_i \leq \gamma_{ck}$'. In other words, γ_{ck} estimates where along

the stimulus dimension the Sigmoid is combined with a Gaussian. To avoid estimation of the peak location at the lower endpoint of the stimulus continuum which would render the Gaussian slope unidentifiable, we truncated the γ_{ck} estimates to be larger or equal in location to stimulus 3. The stimulus values x_i are transformed from 1 to 8 to standardized values ($M = 0$, $SD = 1$) for scaling the parameters where 0 reflects the middle of the stimulus space.

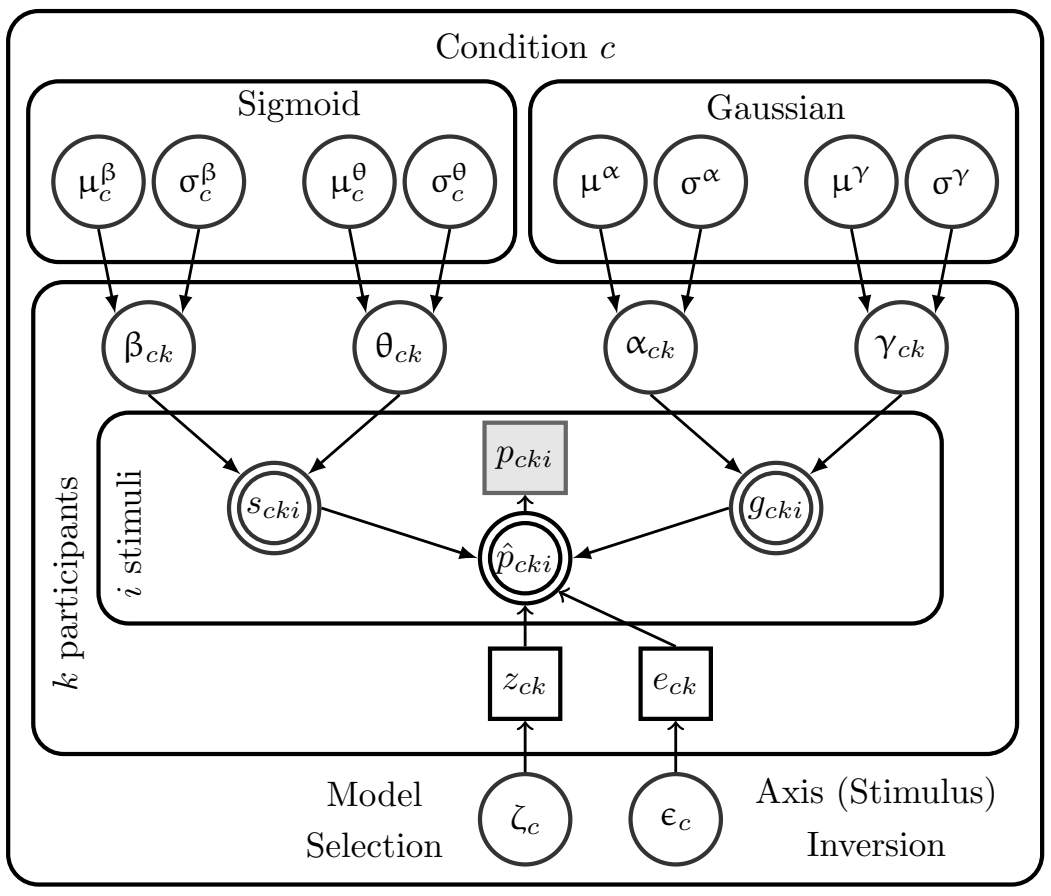

Priors

Sigmoid

$\mu_c^\beta \leftarrow$ MM1

$\mu_c^\theta \leftarrow$ MM2

$\sigma_c^\beta \sim \text{Gaussian}_{[0,2.5]}(1, .1)$

$\sigma_c^\theta \sim \text{Gaussian}_{[0,1]}(1, .1)$

Gaussian

$\mu^\alpha \sim \text{Gaussian}_{[0,1]}(1, 1)$

$\mu^\gamma \sim \text{Gaussian}_{[-1,0]}(-0.61, 1)$

$\sigma^\alpha \sim \text{Gaussian}_{[0,.5]}(1, .1)$

$\sigma^\gamma \leftarrow .25$

Individual Predictions

$\beta_{ck} \leftarrow \mu_c^\beta + \text{Gaussian}_{[-4,4]}(0, 1/(\sigma_c^\beta)^2)$

$\theta_{ck} \leftarrow \mu_c^\theta + \text{Gaussian}_{[-.5,.5]}(0, 1/(\sigma_c^\theta)^2)$

$s_{cki} \leftarrow (e^{\beta_{ck} \cdot (\theta_{ck} - x_i)})^{-1}$

$\alpha_{ck} \sim \text{Gaussian}(\mu_c^\alpha, 1/(\sigma_c^\alpha)^2)$

$\gamma_{ck} \sim \text{Gaussian}(\mu_c^\gamma, 1/(\sigma_c^\gamma)^2)$

$g_{cki} \leftarrow e^{-(\theta_{ck} - x_i)^2 / 2 \cdot \gamma_{ck}^2}$

Model Selection

$\epsilon_c \sim \text{Beta}(1, 1)$

$\zeta_c \leftarrow (1 + e^{\text{MM3}})^{-1}$

$o \sim \text{Uniform}(0, 10)$

$e_{ck} \sim \text{Categorical}(\epsilon_c)$

$z_{ck} \sim \text{Categorical}(\zeta_c \cdot o)$

$$\hat{p}'_{cki} \leftarrow \begin{cases} s_{cki}, \text{ if } z_{ck} = 1 \\ g_{cki} \cdot \min(s_{ck(i<\gamma_{ck})}), \text{ if } z_{ck} = 2 \text{ for } x_i < \gamma_{ck} \end{cases}$$

$\hat{p}_{cki} \leftarrow \hat{p}'_{cki}$, if $e_{ck} = 1$, else invert i

$p_{cki} \sim \text{Binomial}(\hat{p}_{cki}, 10)$

Figure 1

Graphical Bayesian Model for the Mixture of Gaussian vs. Sigmoid Patterns. The terms MM1, MM2, MM3 reflect a Model-Matrix for hypothesis tests (main effects and interaction terms), explained in the text.

In order to seamlessly transition between both function's predictions, the Gaussian prediction g_{cki} is tranformed via $g_{cki} \cdot \min(s_{ck(i \leq \gamma_{ck})})$. That is, the standard Gaussian has by definition a peak-height of 1 but the Sigmoid at the estimated transition location is not necessarily 1. The term min() scales the Gaussian curve at its peak down to the Sigmoid height. Specifically, min() reflects that the Sigmoid by definition can only increase with more extreme values ($i < \gamma_{ck}$), and the minimum is the predicted response strength at the transition location, or in other words, the estimated height of the Gaussian peak, if assigned.

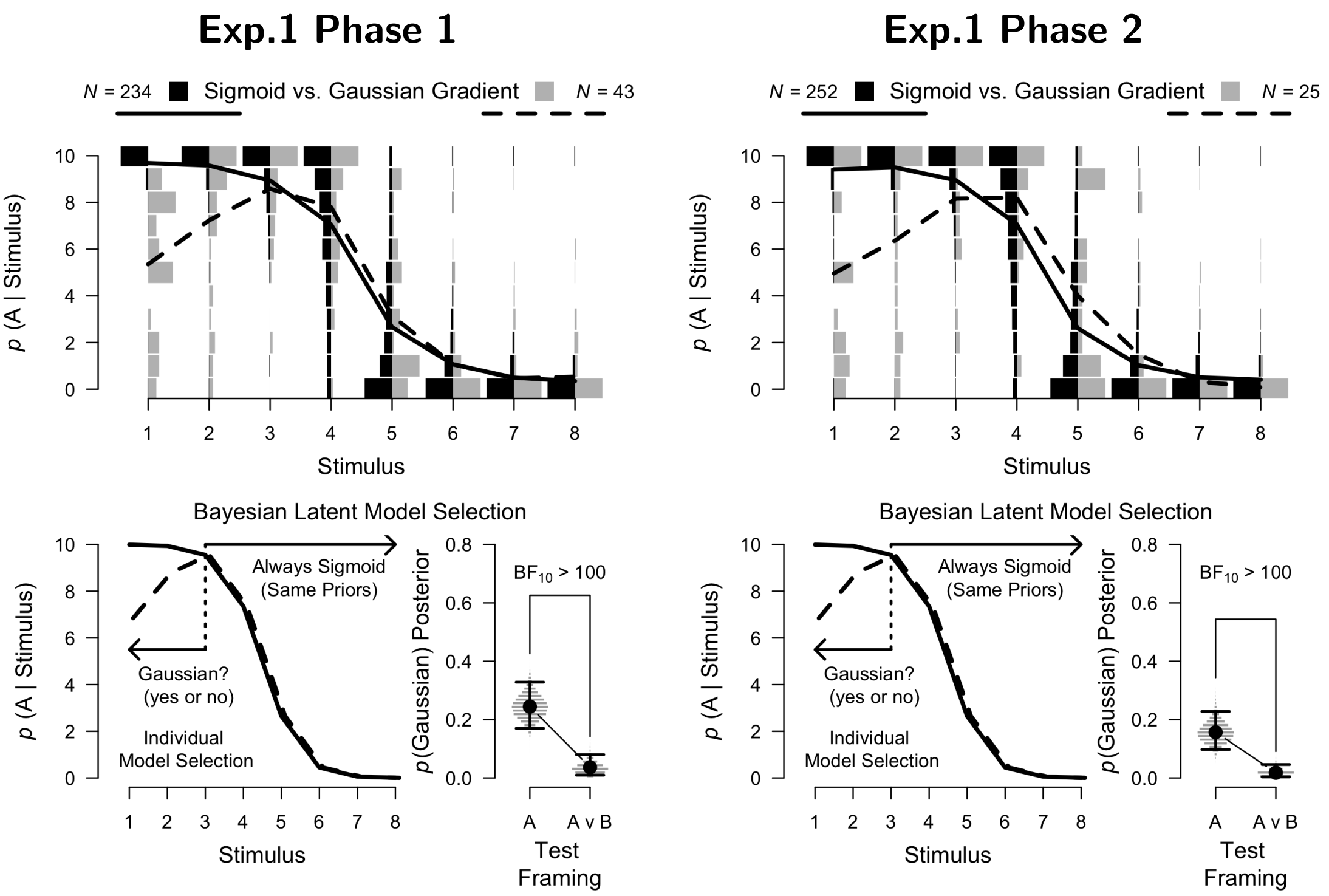

Figure 2

Experiment 1 Model Assignments for both Test Phases. ***Top row:*** *Generalization gradients (response probability on y-axis, stimuli on x-axis) by model assignment. Histograms in background show individual response probabilities, lines depict means.* ***Bottom row:*** *Schematic of Bayesian analysis method as in main manuscript. Stimulus 3 = trained 'A' stimulus. Right-hand side from stimulus 3, sigmoidal function is applied in all cases. Left-hand side Gaussian is augmented in individual model selection.* ***Bottom right:*** *Bayesian posteriors of model selection parameter ζ_c for the main effect of Test Framing.*

The model selection procedure debiases both functions which otherwise would be affected by observations that do not conform with the assumed gradient shape (as shown for unsystematic lapse cases in Sigmoid functions in M. D. Lee & Wagenmakers, 2014, pp. 173f). The model indicator z_{ck} is sampled from ζ_c, which reflects the overall probability of model assignment in the given condition. Thus, z_{ck} can be used to compute the relative number of assignments as within-participant model probability, and ζ_c can be used to test for differences in the average assignments between

experimental conditions. The model in Figure 1 displays a logistic regression, implementing a model-matrix (MM3), reflecting that all main effects and interactions are estimated. The model matrix is identical for the tests on the slope and threshold parameters.

The model matrix was generated using the R base package model.matrix (R Core Team, 2019), and leads to one intercept plus fixed effects (e.g., in Experiment 1: Domain, Framing, Format, Domain:Framing, Domain:Format, Framing:Format, and Domain:Format:Framing), for which we used contrast coding (taking 1 and -1 values) depending on which of the eight design cells the participant was in. We then multiplied these values before summation, with weight-parameters estimated for each effect. The intercept of the slope had the prior $\beta_{\text{int}} \sim \text{Gaussian}(5, 1)$ (i.e., assuming some increase in the gradient over the stimuli on average) and each effect with $\beta_{\text{effect}} \sim \text{Gaussian}_{[-5,5]}(0, 1)$. We estimated the threshold intercept with $\theta_{\text{int}} \sim \text{Gaussian}_{[-2,2]}(0, 1)$ and the effects with the same prior definition. The design-cell means were further estimated hierarchically, by adding an independent error term (σ; by-participant intercepts) at the individual level. We estimated the model selection intercept with $\zeta_{\text{int}} \sim \text{Gaussian}(-1.5, 1)$ and the effects $\zeta_{\text{effect}} \sim \text{Gaussian}(0, 1)$, passing the sum to a logistic function to predict the model probability. In individual model selection (z_{ck}), we multiplied ζ_{effect} with the sample-constant estimate o. This parameter reflects the certainty with which the assignments can be made at the hyper level (i.e., low values with noisy probability, high values with precise probability distribution). We did not apply by-design-cell priors for the Gaussian parameters, as some cells contained few corresponding assignments. This meant that all between-participant variance for the Gaussian parameters loads on the by-participant variance term.

As can be seen in Figure 1, for most priors, we used truncation to restrict the parameter samples to a reasonable space on the log-scale (e.g., values above 15 for β are basically meaningless). Furthermore, the truncations aided in reducing potential model-mimicry. For example, as highlighted above, when the Gaussian α values take values larger than 1 or when γ is estimated to be at stimulus 1, the function becomes

closer to the Sigmoid shape producing issues in identifying the underlying function. Note, we fixed the hierarchical variance for the estimated γ threshold for the individuals to .15, to add uncertainty. Otherwise, in free estimation, the model would be too flexible as it would trade off individual variance in this parameter with the shape of the Sigmoid to adjust in height.

Finally, in a small number of cases, participants' response gradients were apparently flipped from left to right (participants either confused the training associations, or the response buttons at test). To avoid data loss from excluding these participants, we also implemented the selection term ϵ_c which estimates whether the prediction (after the above model selection) should be applied to the participant's data as is, or whether it should be flipped along the stimulus axis. For this, we simply exchanged the predictions between stimulus 1 with 8, 2 with 7, 3 with 6, with 4 with 5, otherwise, all model definitions applied as defined.

In Experiment 1, the above-described model was pre-registered and applied to the data. For Experiment 2, we pre-registered this model as well as a potential necessary adjustment (highlighted in the main article), which we ultimately implemented. That is, we originally assumed that the peak location should be centered near the stimulus that was trained together with the currently queried test category (i.e., at stimulus 3 for A and stimulus 6 for NoA, see Figure 2). However, in Experiment 2, we anticipated that rendering a judgment for 'No A' might be processed as the *negation* of 'A'. This requires participants to invert their estimate about the likelihood of the A outcome/category in order to provide a judgment about the likelihood of NoA (i.e., estimate(NoA)= 1-estimate(A)). Thus in Experiment 2, we included an additional qualitative parameter (in addition to p[Gaussian]) that estimates whether the NoA gradient should be treated as the negation of A vs independent of A (henceforth negation model).

Fortunately, the negation model can be easily implemented as a characteristic of the data structure, leaving all other specifications untouched. In formal notation, let the default be model y=1, and the negation model y=2. With model 1 we arrange the data such that we center the gradient peak on the training exemplar of the test-queried

category. This is achieved by flipping the NoA gradient over the x-axis, thereby aligning the gradient peak at the same location as the A gradient. With model y=2, we flip both the x- and y-axes (see Figure 4 in main text). Prior to this flipping, we first estimated the shape of the A gradients, and only then estimated whether model 1 or 2 is better at fitting the noA gradient (since classification of the noA gradient is dependent on the A gradient). If model y=1 won, then we assumed the same shape (p[Gaussian], but allowing condition-specific parameters [slope, threshold]). The model selection was estimated by the individual model indicator $\upsilon_{kc} \sim \text{Cat}(\text{MM4})$. The model matrix MM4 was specified in the same way as the other model matrices.

Experiment 1

Experiment 1 included two additional within-participant test phases for counterbalancing. After test phase 1 (reported in the main article), participants were given the same series of tests but using the alternative response Format (i.e., Ratings after repeated Decisions, or vice versa), while keeping the Framing consistent (since this was manipulated between-subjects). A final (third) test phase asked participants to provide judgments for the alternative outcome or category (i.e. 'Bugi' in categorization, 'no Agog' in prediction) using a single format (absolute ratings).

Test Phase 2

We conducted the same set of analyses for phase 2 as we did for phase 1 as per the main manuscript. Table 1 shows the test results for all main effects and interactions, and Figures 3 and 4 show the gradients and behavioral trends. As can be seen in Table 1, all effects reported for the model assignment and the slope parameter in test Phase 1 (main manuscript) replicate in Phase 2, with the exception of the two-way interaction between Format and Framing, and the three-way interaction with Domain. Figure 4A shows that main effect of Framing on p(Gaussian) seen in phase 1 was also apparent in phase 2, despite Gaussian assignments being less frequent overall. In addition, Figure 3B illustrates that the lower slope for Absolute Decisions in Categorization became less pronounced compared to phase 1. Thus, taking both test phases together, it seems that category judgments are less steep at the boundary compared to predictive judgments in

Decisions, but the opposite occurs with the Rating Format (two-way interaction of Format and Domain).

Additionally, Figure 3 (right panels) shows that when Ratings were performed in Phase 2, the gradients became flatter compared to all other conditions. This likely reflects unsupervised learning trends, since those participants performed 80 decision trials prior to these ratings (Livesey & McLaren, 2009; Lovibond et al., 2020). These results suggest that testing effects should be considered when designing lengthy test phases to assess generalization, even under conditions where feedback is withheld (see J. C. Lee et al., 2022).

Table 1

Bayes Factors for effects for each parameter in Experiment 1, Test Phase 2.

**Effect**	**Qualitative Parameters**	**Quantitative Parameters**	
	p(Gaussian)	slope	midpoint
Domain	0.596	0.174	0.098
Format	0.393	>100	0.091
Framing	>100	0.376	0.017
Domain x Format	0.345	46.062	0.015
Domain x Framing	1.501	0.16	0.216
Format x Framing	0.396	0.331	0.018
Domain x Format x Framing	0.361	0.19	0.02

Note. List of factors: learning Domain (Categorization vs Prediction), test Format (binary Decisions vs continuous Ratings), test Framing (Relative vs Absolute).

Test Phase 3

Finally, we included an exploratory test phase to assess what participants learned about the alternative No Agog outcome in Prediction, and the alternative Bugi outcome in Categorization (using Absolute Ratings only). We speculated that Prediction participants would primarily learn about the observed outcome and neglect the absent outcome, while Categorization participants might learn about both categories.

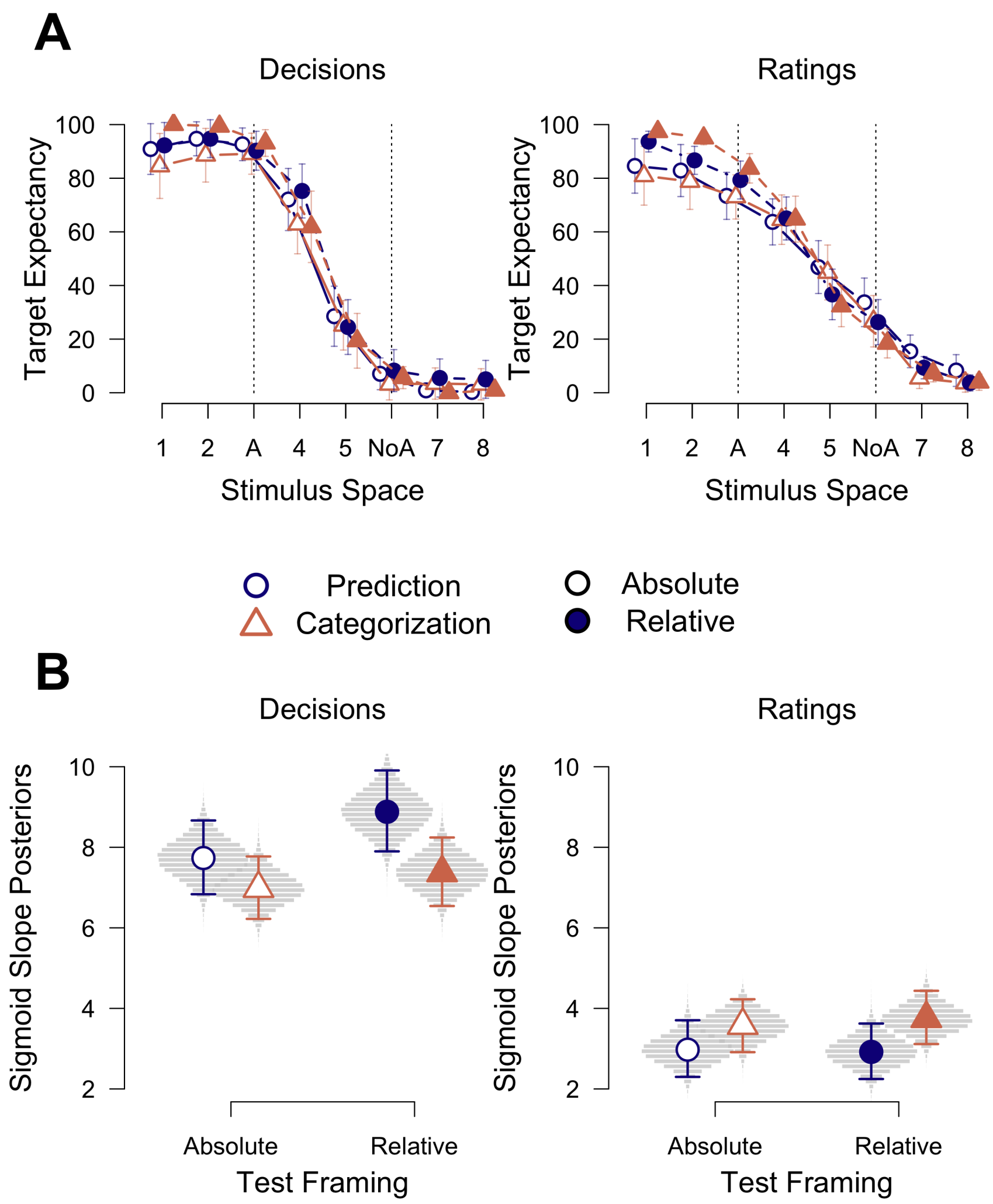

Figure 3

A) *Generalization gradients for each condition in test phase 2 in Experiment 1 (N = 277).* ***B)*** *Posteriors for the slope parameter for each condition in test phase 2 in Experiment 1.*

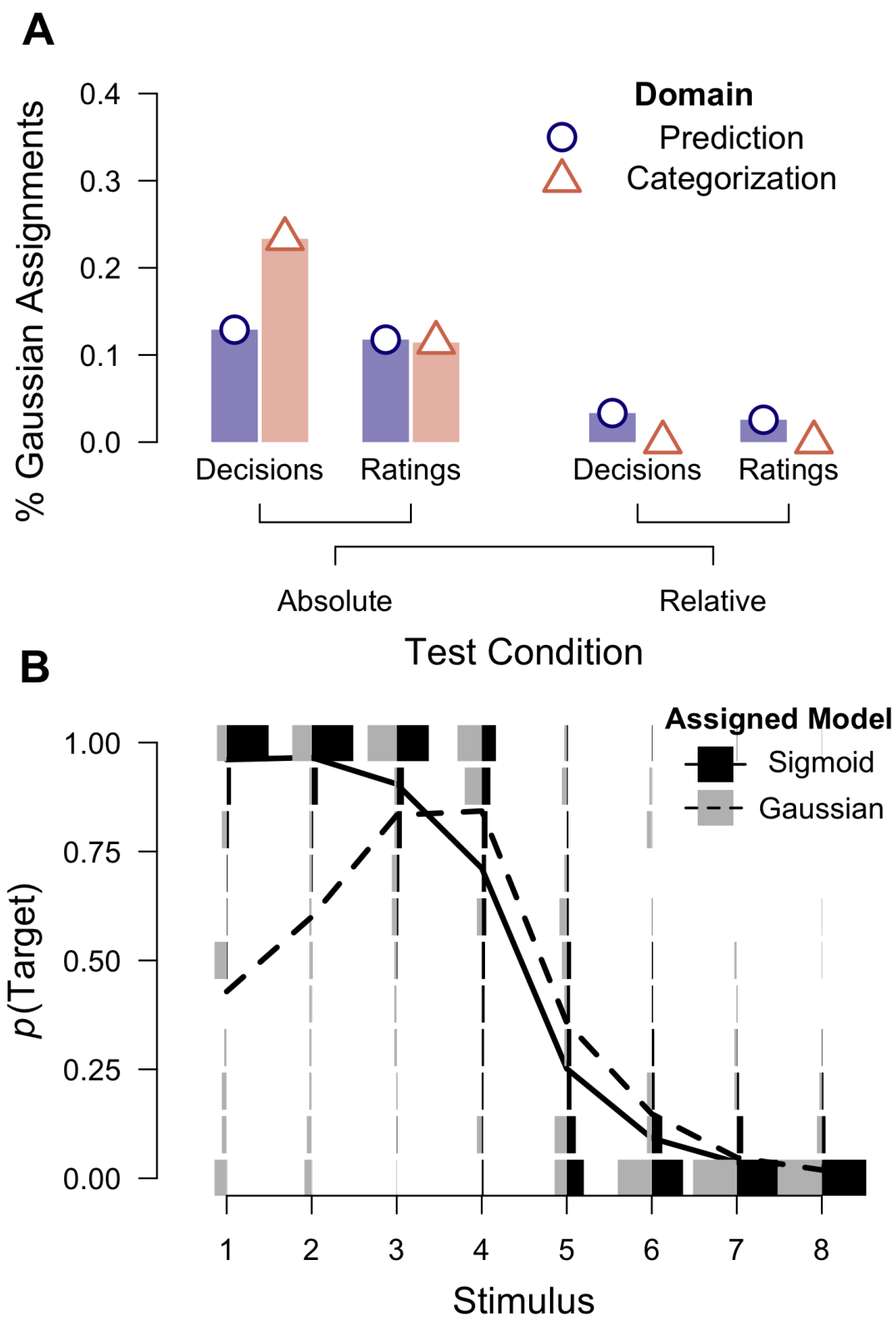

Figure 4

A) *Proportion of participants in each condition in Experiment 1 (Phase 2) assigned to the Gaussian model with >80% confidence.* ***B)*** *Generalization gradients for each assigned model in Experiment 1 (total* $N = 265$ *of* $N = 277$*, with* $N = 34$ *assigned to Gaussian). Histograms reflect distribution of responses.*

Consequently, when asking to rate the likelihood of No Agog (ranging from 0% to 100%), the gradients might be flatter due compared to rating the likelihood of (category) Bugi.

Figure 5 plots the resulting ratings and slope estimates for the alternative outcome (blue), for Prediction (right) and Categorization (left) domains. The flatter gradient for Prediction suggests that participants found rating the likelihood of the No Agog outcome much more difficult than the Bugi category. As mentioned above, this could be because participants learned stimulus-category associations with Bugi in Categorization, but did not learn associations with No Agog in Prediction. An alternative interpretation is that the response scale involved negating an already

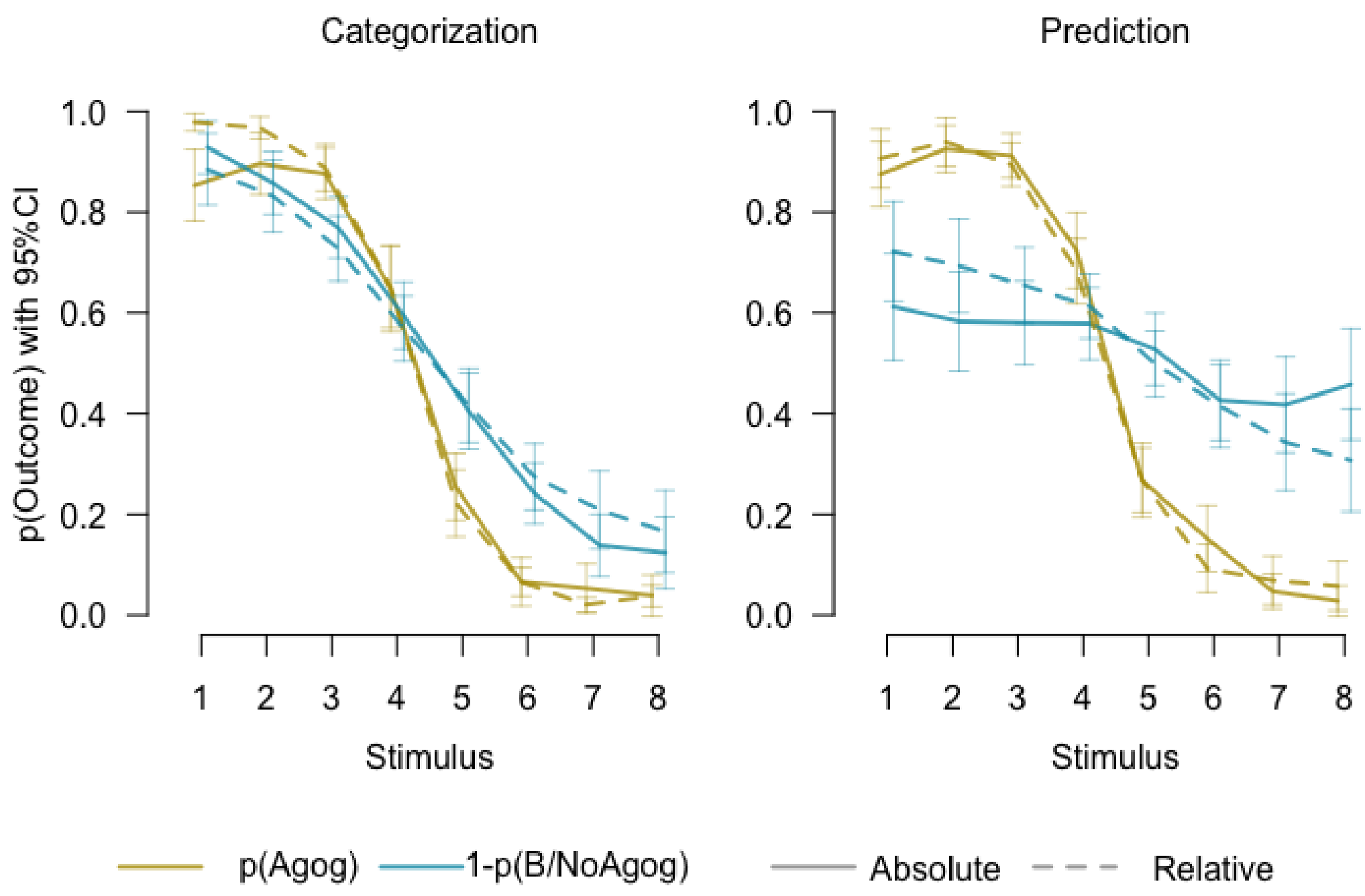

Figure 5

Absolute ratings for the Agog outcome/category (yellow) in Test Phases 1/2 (collapsed) and the alternative outcome/category (blue, Bugi in Categorization, No Agog in Prediction) in Test Phase 3 in Experiment 1. Line types refer to Framing condition.

negated outcome (i.e., 0% No Agog) which may have caused confusion and encouraged responding toward the midpoint of the scale. The question of whether these gradients depended on the training Domain or the (confounded) outcome labels presented in training (Bugi in Categorization and No Agog in Prediction) inspired the design of Experiment 2 as reported in the main manuscript.

Interestingly, the task for Prediction participants appeared to be less difficult after test phases where participants gave Relative judgments (i.e., compared Agog vs No Agog as opposed to rating the likelihood of the Agog outcome alone). This may be because relative judgments encourage participants to think of the absent outcome as an explicit possibility in and of itself. We note this result for interest and are hesitant to speculate further about this result since it was not the focus of our study. It would be interesting for future studies to investigate how representation of present and absent outcomes differ, and how these representations interact with testing format to

determine generalization.

References

Hanson, H. M. (1959). Effects of discrimination training on stimulus generalization. *Journal of Experimental Psychology*, *58*(5), 321–334. https://doi.org/10.1037/h0042606

Lee, J. C., Le Pelley, M. E., & Lovibond, P. F. (2022). Nonreactive testing: Evaluating the effect of withholding feedback in predictive learning. *Journal of Experimental Psychology: Animal Learning and Cognition*, *48*(1), 17–28. https://doi.org/10.1037/xan0000311

Lee, J. C., Mills, L., Hayes, B. K., & Livesey, E. J. (2021). Modelling generalisation gradients as augmented gaussian functions. *Quarterly Journal of Experimental Psychology*, *74*(1), 106–121. https://doi.org/10.1177/1747021820918857

Lee, M. D., & Wagenmakers, E.-J. (2014). *Bayesian cognitive modeling: A practical course*. Cambridge University Press. https://doi.org/10.1017/CBO9781139087759

Livesey, E. J., & McLaren, I. P. L. (2009). Discrimination and generalization along a simple dimension: Peak shift and rule-governed responding. *Journal of Experimental Psychology: Animal Behavior Processes*, *35*(4), 554–565. https://doi.org/10.1037/a0015524

Lovibond, P. F., Lee, J. C., & Hayes, B. K. (2020). Stimulus discriminability and induction as independent components of generalization. *Journal of Experimental Psychology: Learning, Memory, and Cognition*, *46*(6), 1106–1120. https://doi.org/10.1037/xlm0000779

R Core Team. (2019). *R: A language and environment for statistical computing*. R Foundation for Statistical Computing. Vienna, Austria. https://www.R-project.org/

Sisson, S. A. (2005). Transdimensional Markov chains: A decade of progress and future perspectives. *Journal of the American Statistical Association*, *100*(471), 1077–1089. https://doi.org/10.1198/016214505000000664
